# Properties of multiwinner voting rules

**DOI:** 10.1007/s00355-017-1026-z

**Published:** 2017-01-30

**Authors:** Edith Elkind, Piotr Faliszewski, Piotr Skowron, Arkadii Slinko

**Affiliations:** 1 University of Oxford, Oxford, UK; 2 AGH University, Krakow, Poland; 3 University of Auckland, Auckland, New Zealand

## Abstract

A committee selection rule (or, multiwinner voting rule) is a mapping that takes a collection of strict preference rankings and a positive integer *k* as input, and outputs one or more subsets of candidates of size *k*. In this paper we consider committee selection rules that can be viewed as generalizations of single-winner scoring rules, including SNTV, Bloc, *k*-Borda, STV, as well as several variants of the Chamberlin–Courant rule and the Monroe rule and their approximations. We identify two natural broad classes of committee selection rules, and show that many of the existing rules belong to one or both of these classes. We then formulate a number of desirable properties of committee selection rules, and evaluate the rules we consider with respect to these properties.

## Introduction

There are many situations where a society needs to select a subset of a given size from the set of available options. For example, in indirect democracies people choose representatives to govern on their behalf, companies select groups of products to promote to their customers (Lu and Boutilier 2011, 2015; Skowron et al. 2016), search engines decide which webpages to display in response to a given query (Dwork et al. 2001), and among many applicants for a job (e.g., a tenure-track position at a university) several get short-listed and invited for interviews. For all these tasks we need formal rules to perform the selection, and the desirable properties of such rules may depend on the task at hand. We view these selection rules as multiwinner voting rules which, given individual preferences and the desired number of winners, output groups of winners, which we call *committees*.

Multiwinner elections are even more ubiquitous than single-winner ones, but much less studied. They were implicitly considered under the umbrella of choice functions (Arrow 1951; Fishburn 1973), but in this model the size of the elected committee could not be controlled. It was Debord (1993) and Felsenthal and Maoz (1992) who introduced several *k*-choice functions that elect committees of size exactly *k*, and investigated their properties. However, even within the space of rules that elect exactly *k* winners, many distinct models can happily coexist. One way to classify them is according to the type of the input: there are preference-based rules (whose inputs are sequences of linear orders; see, e.g., the work of Brams and Fishburn (2002)), approval-based rules (whose inputs are sequences of dichotomies; see, e.g., the works of Brams et al. (2007), Caragiannis et al. (2010), Kilgour and Marshall (2012), Aziz et al. (2017), and the overview by Kilgour (2010)), and tournament-based rules (whose inputs are either tournaments or weighted tournaments; see the book of Laslier (1997) for a general overview of tournament-based rules). Also, a number of authors consider settings where the compatibility of committee members is an important issue, and therefore voters’ preferences over committees cannot be deduced from their preferences over individual alternatives alone (Ratliff 2006; Uckelman 2010; Ratliff and Saari 2014). For a different perspective on this issue, see the line of work on voting in combinatorial domains ( Lang and Xia (2016) offer a very good overview of this area).

In this work, we focus on the model where the goal is to select a committee of a given size on the basis of voters’ ordinal preferences over the candidates. A number of rules for this task are based on various forms of the Condorcet principle (Fishburn 1981a, b; Gehrlein 1985; Kaymak and Sanver 2003; Ratliff 2003; Barberà and Coelho 2008; Elkind et al. 2015). In contrast, we consider rules that, informally speaking, can be seen as counterparts of positional scoring rules. Our aim is to present a uniform framework for the study of such rules, to identify useful classes of score-based committee selection rules, and to propose a set of natural properties (axioms) against which multiwinner rules can be judged.

We have selected ten voting rules as examples of different ideas pertaining to score-based committee selection: STV, SNTV, *k*-Borda, Bloc, three variants of the Chamberlin–Courant rule (Chamberlin and Courant 1983; Lu and Boutilier 2011; Betzler et al. 2013), and three variants of the Monroe rule (Monroe 1995; Betzler et al. 2013; Skowron et al. 2015). STV and SNTV are well-known rules that are used for parliamentary elections in several countries;^1^ Bloc is a rule that asks each voter to specify her favourite committee of *k* candidates and selects *k* candidates that were mentioned more frequently than others; *k*-Borda picks *k* alternatives with the highest Borda scores and is representative of rules used for choosing *k* finalists in a competition (indeed, Formula 1 racing and the Eurovision song contest use scoring rules very similar to Borda). The Chamberlin–Courant and Monroe rules are examples of rules that, like STV, focus on proportional representation, but, unlike STV, explicitly assign a committee member (a representative) to each voter. We also consider two rules that are based on approximation algorithms for, respectively, the Chamberlin–Courant rule (Lu and Boutilier 2011) and the Monroe rule (Skowron et al. 2015); that is, we treat these algorithms as voting rules in their own right. All these rules can be seen as being loosely based on single-winner scoring rules.

We are interested in judging the committee selection rules on our list with respect to their applicability in the following settings:

- **Parliamentary elections** Voting rules for such elections should respect the “one person, one vote” principle. This is reflected in the requirement that each elected member should represent approximately the same number of voters. Some such rules make use of electoral districts, i.e., they are based on holding separate (possibly multiwinner) elections in different parts of the country, while others treat the whole country as a single constituency, and focus on proportional representation of different population groups or political parties.
- **Shortlisting** Consider a situation where a position is filled at a university. Each faculty member ranks applicants in order to create a short-list of those to be invited for an interview. One of the important requirements in this case is that if some candidate is shortlisted when *k* applicants are selected, then this candidate should also be shortlisted if the list is extended to $k+1$ applicants.
- **Movie selection** Based on rankings provided by different customer groups, an airline has to decide which (few) movies to offer on their long-distance flights. It is important that each passenger finds something satisfying to watch. This task is similar to parliamentary elections, but without the need to ensure that each movie would be watched by the same number of people. It is, however, quite different from shortlisting: If there are two similar job candidates, then it is desirable to interview either both of them or neither of them, whereas if two movies are similar, it makes sense to pick at most one of them. Skowron et al. (2016) discuss this view of multiwinner elections and provide a number of other examples and applications.

We study properties of voting rules that are relevant to the above-listed settings. Specifically, we discuss properties that are specific to the task of committee selection, such as committee monotonicity, the solid coalitions property, and the consensus committee property, and adapt the standard notions of unanimity, monotonicity, homogeneity, and consistency to the multiwinner setting. For every such property $\mathcal{P}$ and each rule $\mathcal{R}$ considered in this paper, we determine if $\mathcal{R}$ satisfies $\mathcal{P}$ (sometimes we need to place mild technical conditions on the input, e.g., a rule may satisfy a certain property only if the target committee size divides the number of voters or if the number of voters is sufficiently large). Our results are summarized in Table 1 (see Sect. 10).

In the course of our investigation, we identify an important class of committee selection rules, which we call *committee scoring rules*. Within the class of committee scoring rules, we distinguish *(weakly) separable rules* and *representation-focused rules*. These notions prove to be useful for our analysis, as some axioms turn out to be satisfied by all rules that belong to one of these subclasses, or by an easily identifiable subset of such rules (see, e.g., Theorem 3). Further, we obtain a simple and intuitive characterization of separable committee scoring rules. Namely, we show that all such rules are *best-k* rules; roughly speaking, this means that each such rule first ranks all candidates and then outputs the top *k* candidates according to this ranking. We believe that introducing the class of committee scoring rules is one of the most important contributions of this paper. In a series of follow-up papers, Faliszewski et al. conduct a more detailed study of the internal structure of this class of rules (Faliszewski et al. 2016a, b) and Skowron et al. (2016b) provide an axiomatic characterization of such rules.

Our paper is a preliminary attempt to develop a formal framework for the study of preference-based committee selection rules. Thus we use the word *axiom* quite freely, without implying that our axioms should be viewed as *normative requirements*. Further, our work focuses on committee selection rules that are based on scoring procedures. Such rules are fundamentally different from those that are motivated by the Condorcet criterion. In particular, in the single-winner case, no scoring rule is Condorcet consistent; consequently, a committee selection rule that reduces to a scoring rule for the case when the target committee size equals one cannot satisfy any reasonable extension of the Condorcet criterion.^2^ It would be very interesting to apply our framework to Condorcet-based rules. However, this task is beyond the scope of this paper, and we leave it as future work.

The remainder of the paper is organized as follows. In Sect. 2 we introduce the basic terminology used throughout the paper; in Sect. 3 we define the rules that we study and put forward two ways of classifying them. In Sect. 4, we define several properties of committee selection rules, and in Sects. 5–8 we study particular groups of these properties in detail. We discuss related literature in Sect. 9 and conclude in Sect. 10.

## Preliminaries

An *election* is a pair $E = (C,V)$, where $C = \{c_1, \ldots , c_m\}$ is a set of *candidates*, or *alternatives* (we will use these two terms interchangeably), and $V = (v_1, \ldots , v_n)$ is a list of *voters*. For succinctness, each voter is identified with her *preference order*, i.e., a ranking of the candidates from the most desirable one to the least desirable one. That is, $v_i$ refers both to the *i*-th voter in *V* and the preference order of that voter. We write $a\succ _i b$ or $v_i:a\succ b$ when *a* is ranked above *b* in $v_i$. We write $\Vert C\Vert$ and $\Vert V\Vert$ to denote the size of the set *C* and the length of the list *V*, respectively. Also, for readability, we use set-theoretic notation when we reason about *V*: e.g., we write $V'\subseteq V$ when $V'$ can be obtained from *V* by deleting some of the voters. We denote the position of a candidate $c\in C$ in vote $v\in V$ by ${{{\mathrm {pos}}}}_v(c)$. If $V_1$ and $V_2$ are two lists of voters over the same candidate set *C*, then $V_1+V_2$ denotes the concatenation of $V_1$ and $V_2$. If *V* is a list of voters and *t* is an integer, then *tV* denotes the concatenation of *t* copies of *V*. For $E_1 = (C,V_1)$ and $E_2 = (C,V_2)$, we write $E_1+E_2$ to denote $(C,V_1+V_2)$, and for $E = (C,V)$ and a positive integer *t*, we write *tE* to denote (*C*, *tV*). For an integer *n*, we denote $\{1, \ldots , n\}$ by [*n*].

A *committee selection rule* (or, a *multiwinner rule*) ${{\mathcal {R}}}$ is a function that, given an election $E = (C,V)$ and a positive integer *k*, $k \le \Vert C\Vert$, returns a non-empty set ${{\mathcal {R}}}(E,k)$ of *k*-element subsets of *C*, which we call *committees*. That is, a rule returns the set of committees that are tied for winning. In practice, one would need to combine such a rule with a tie-breaking mechanism, but for simplicity we mostly disregard this issue here. Brams and Fishburn (2002) introduced *choose-k* rules, but their definition stipulates that such a rule selects *at least* *k* alternatives. Two early papers that focus on rules selecting committees of size *exactly* *k* are those of Debord (1993) and of Felsenthal and Maoz (1992).

Requiring committee selection rules to pick fixed-size committees is natural if, for example, the goal is to elect a parliament whose size is fixed by the constitution. However, as a consequence, we are sometimes forced to elect committees that are Pareto dominated under reasonable preference extensions: e.g., if in each vote the candidates are ranked as $a\succ b\succ c \succ d$ and $k=2$, the voters may prefer $\{a\}$ to $\{a, b\}$, but selecting *a* alone is not possible in our model. Alternatively, we could require ${{\mathcal {R}}}(E,k)$ to return committees of size at most *k*. The latter approach is also studied in the literature (either explicitly or implicitly), but we adopt the former one due to its simplicity and applicability in our settings of interest.

## Committee selection rules

We now provide definitions of the committee selection rules that we study and discuss two general ways of defining such rules.

### Common committee selection rules

A number of committee selection rules are inspired by single-winner rules, so let us review these first. Many single-winner rules calculate the scores of alternatives in order to decide which one is the best. Here are some popular ways of computing the candidates’ scores.

- **Plurality score** The *Plurality score* of a candidate *c* is the number of votes where *c* is ranked first.
- ${\mathbf {t}}$ **-approval score** Let *t* be a positive integer. The *t*-*approval score* of a candidate *c* is the number of votes where *c* is ranked in top *t* positions.
- **Borda score** Let *v* be a vote in an election (*C*, *V*). The Borda score that candidate $c\in C$ receives from *v* is $\Vert C\Vert -{{{\mathrm {pos}}}}_v(c)$. The *Borda score* of *c* in (*C*, *V*) is the sum of *c*’s Borda scores from all votes in *V*.
- **s-score** The three types of scores listed above are special cases of the following general framework. Consider a setting with a candidate set *C*, $\Vert C\Vert =m$, and a score vector $\mathbf{s}=(s_1, \ldots , s_m)$, where $s_1\ge s_2\ge \cdots \ge s_m$ and $s_1>s_m$. We define the $\mathbf{s}$-*score* of a candidate *a* in an election (*C*, *V*) with $V = (v_1,\ldots ,v_n)$ as
  $$\begin{aligned} \text {sc}_\mathbf{s}(a)= \sum _{i=1}^n s_{\text {pos}_{v_i}(a)}. \end{aligned}$$
  It is then immediate that the Plurality score is the $(1,0, \ldots , 0)$-score, the *t*-approval score is the $(\underbrace{1,\ldots ,1}_t,0,\ldots ,0)$-score, and the Borda score is the $(m-1, m-2, \ldots , 0)$-score.

Given these definitions, we are ready to describe the committee selection rules that we focus on in this paper. Let $E = (C,V)$ be an election and let $k \in [\Vert C\Vert ]$ be the target committee size. We assume the parallel-universes tie-breaking (Conitzer et al. 2009), i.e., each of our rules returns all the committees that could result from breaking the ties that arise during the application of the rule.

- **Single transferable vote (STV)** STV is a multistage elimination rule that works as follows. In each stage, if there is a candidate *c* whose Plurality score is at least $q = \left\lfloor \frac{\Vert V\Vert }{k+1} \right\rfloor + 1$ (the so-called Droop quota), we do the following: (a) include *c* in the winning committee, (b) delete *q* votes where *c* is ranked first, so that each of these votes is ‘transferred’ to the candidate currently ranked right after *c*, and (c) remove *c* from all the remaining votes. If each candidate’s Plurality score is less than *q*, a candidate with the lowest Plurality score is deleted from all votes. We repeat this process until *k* candidates are selected. Note that parallel-universes tie-breaking requires us to consider all possible ways to select a candidate among those whose score meets or exceeds the quota, to identify *q* votes to be deleted among those where this candidate is ranked first (note that this determines the ‘transfers’, i.e., how many extra votes will be gained by each surviving candidate), and to pick a candidate to be eliminated among those with the lowest score; a committee wins under STV if it can be obtained by the procedure described above for *some* sequence of such choices. There are also many other variants of STV; we point the reader to the work of Tideman and Richardson (2000) for details. In particular, the reader can verify that all results in our paper continue to hold if we require that the number of the ‘extra’ votes of a selected candidate *c* that are transferred to another candidate *a* is proportional to the number of votes of the form $c\succ a\succ \cdots$ at the respective stage.
- **Single nontransferable vote (SNTV)** SNTV returns *k* candidates with the highest Plurality scores (thus one can think of SNTV as simply *k*-Plurality).
- **Bloc** Bloc returns *k* candidates with the highest *k*-approval scores. Observe that by using *k*-approval where *k* is the target committee size, we ensure that when all voters rank the candidates in the same way, the unique winning committee consists of the candidates ranked in top *k* positions by all the voters (whereas for *s*-approval with $s\ne k$ this is not the case).
- ${\mathbf {k}}$ **-Borda** *k*-Borda returns *k* candidates with the highest Borda scores.
- **The Chamberlin–Courant and Monroe rules** These rules explicitly aim at proportional representation. The main idea is to provide an optimal assignment of committee members to voters, by using a satisfaction function to measure the quality of the assignment. A *satisfaction function* is a nonincreasing mapping $\alpha :{{\mathbb {N}}} \rightarrow {{\mathbb {N}}}$. Intuitively, $\alpha (i)$ is a voter’s satisfaction from being represented by a candidate that this voter ranks in position *i*. We primarily focus on the Borda satisfaction function, which for *m* candidates is defined as $\alpha ^m_{{{{\beta }}}}(i) = m-i$. A function $\varPhi :V \rightarrow C$ is called an *assignment function*. We write $\varPhi (V)=\{\varPhi (v)\mid v\in V\}$. We say that $\varPhi$ is a *k*-*assignment function* if $\Vert \varPhi (V)\Vert \le k$. Intuitively, $\varPhi (V)$ is the elected committee where voter *v* is represented by candidate $\varPhi (v)$. There are several ways to compute the societal satisfaction from an assignment; we focus on the following two:
  $$\begin{aligned} \ell _1(\varPhi ) = \sum _{v \in V} \alpha ( {{{\mathrm {pos}}}}_v(\varPhi (v)) ), \quad \ell _{\min }(\varPhi ) = \min _{v \in V}\left\{ \alpha ({{{\mathrm {pos}}}}_v(\varPhi (v)) \right\} , \end{aligned}$$
  where $\alpha$ is the given satisfaction function. The former one, $\ell _1(\varPhi )$, is a utilitarian measure, which sums the satisfactions of all the voters, and the latter one, $\ell _{\min }(\varPhi )$, is an egalitarian measure, which considers the satisfaction of the least satisfied voter.
  Let $\alpha$ be a satisfaction function and let $\ell$ be $\ell _1$ or $\ell _{\min }$. The Chamberlin–Courant rule with parameters $\ell$ and $\alpha$ ($\ell$-$\alpha$-CC) finds a *k*-assignment function $\varPhi$ that maximizes $\ell (\varPhi )$ and declares the candidates in $\varPhi (V)$ to be a winning committee. If $\Vert \varPhi (V)\Vert < k$, the rule fills in the missing committee members in an arbitrary way and outputs all resulting committees. Note that this class of rules includes SNTV: indeed, SNTV is exactly $\ell _1$-$\alpha _1$-CC, for satisfaction function $\alpha _1$ given by $\alpha _1(i)=1$ if $i=1$ and $\alpha _1(i)=0$ if $i>1$. The $\ell$-$\alpha$-Monroe rule is defined similarly to $\ell$-$\alpha$-CC, except that we optimize over *k*-assignment functions that additionally satisfy the so-called Monroe criterion, which requires that exactly *k* candidates are elected and each elected candidate is assigned to either $\lfloor \frac{n}{k} \rfloor$ or $\lceil \frac{n}{k} \rceil$ voters. To simplify notation, we omit $\alpha ^m_{{{{\beta }}}}$ when referring to the Monroe/CC rule with the Borda satisfaction function.
  For the Chamberlin–Courant rule, for each set of candidates $C' \subseteq C$ we define the assignment function $\varPhi ^{{{\mathrm {CC}}}}(C'):V\rightarrow C'$ as follows: for each voter *v* the candidate $\varPhi ^{{{\mathrm {CC}}}}(C')(v)$ is *v*’s top candidate in $C'$. If *W* is a winning committee under the Chamberlin–Courant rule, then $\varPhi ^{{{\mathrm {CC}}}}(W)$ is an optimal assignment function.
  The utilitarian variants of these rules, i.e., $\ell _1$-CC and $\ell _1$-Monroe, were introduced by Chamberlin and Courant (1983) and by Monroe (1995), respectively; similar ideas are discussed by Sugden (1984) in a game-theoretic context. The egalitarian variants were introduced by Betzler et al. (2013). Unfortunately, these rules are hard to compute, irrespective of tie-breaking, both for the Borda satisfaction function (Lu and Boutilier 2011; Betzler et al. 2013) and for various approval-based satisfaction functions (Procaccia et al. 2008; Betzler et al. 2013).

- **Approximate variants of** ${\varvec{\ell }_\mathbf{1}}$-**Monroe and** ${\varvec{\ell }_\mathbf{1}}$-**CC** Complexity results for $\ell _1$-CC and $\ell _1$-Monroe inspired research on designing efficient approximation algorithms for these rules. Here, in the spirit of Caragiannis et al. (2014), we consider these algorithms as full-fledged multiwinner rules. We refer to the rules based on approximation algorithms for $\ell _1$-CC and $\ell _1$-Monroe as Greedy-CC and Greedy-Monroe, respectively. Greedy-CC was proposed by Lu and Boutilier (2011) and Greedy-Monroe by Skowron et al. (2015). Both rules use the Borda satisfaction function, aggregated in the utilitarian way (i.e., by using $\ell _1$). They proceed in *k* iterations; in the *i*-th iteration, $i\in [k]$, they build a set $W_i$ of cardinality *i* so that $\emptyset = W_0 \subset W_1 \subset W_2 \subset \cdots \subset W_k$. The set $W_k$ is declared to be the winning committee. In the *i*-th iteration, $i\in [k]$, Greedy-CC picks a candidate $c_i \in C {\setminus } W_{i-1}$ that maximizes $\ell _1(\varPhi ^{{{\mathrm {CC}}}}(W_{i-1}\cup \{c_i\}))$, and sets $W_i = W_{i-1} \cup \{c_i\}$. In particular, $c_1$ is an alternative with the highest Borda score.
  Greedy-Monroe, in addition to the sets $W_0, \ldots , W_k$, also maintains lists of voters $\emptyset = V_0 \subset V_1 \subset \cdots \subset V_k = V$. The list $V_i$, $i\in [k]$, consists of voters that already have candidates assigned to them after the *i*-th iteration. In the *i*-th iteration, $i\in [k]$, the rule picks a number $n_i \in \{\lceil \frac{n}{k}\rceil , \lfloor \frac{n}{k}\rfloor \}$ (see below for the choice criterion) and then picks a candidate $c_i \in C {\setminus } W_{i-1}$ and a group $V'$ of $n_i$ voters from $V{\setminus } V_{i-1}$ that jointly maximize the Borda score of $c_i$ in $V'$. The rule sets $W_i = W_{i-1}\cup \{c_i\}$ and $V_i = V_{i-1} \cup V'$ (intuitively, Greedy-Monroe assigns $c_i$ to the voters in $V'$). Regarding the choice of $n_i$, if *n* is of the form $kn'+n''$, where $0 \le n'' < k$, then Greedy-Monroe sets $n_i$ to $\lceil \frac{n}{k}\rceil$ for $i=1, \ldots , n''$ and to $\lfloor \frac{n}{k}\rfloor$ for $i=n''+1, \ldots , k$. For instance, $c_1$ and $V_1$ are chosen so that for every candidate $c\in C$ and every group of voters $V'\subseteq V$ of size $n_1$ it holds that the Borda score of $c_1$ in $(C, V_1)$ is at least as high as the Borda score of *c* in $(C, V')$ (and, in particular, $c_1$ is a Borda winner in $(C, V_1)$).
  Greedy-CC and Greedy-Monroe output committees that approximately satisfy the optimality criteria of $\ell _1$-CC and $\ell _1$-Monroe. In particular, Greedy-CC finds a committee *W* such that the satisfaction of the voters $\ell _1(\varPhi ^{{{\mathrm {CC}}}}(W))$ is at least $1-\frac{1}{e}$ of the satisfaction achieved under $\ell _1$-CC (Lu and Boutilier 2011). Greedy-Monroe finds a committee that achieves at least a $1-\frac{k}{2m-1} - \frac{H_k}{k}$ fraction of the satisfaction given by $\ell _1$-Monroe, where $H_k=\sum _{i=1}^{k}\frac{1}{k}$ (Skowron et al. 2015); for many practical settings, this value is quite close to 1.

Intuitively, $\ell _1$-CC and $\ell _{\min }$-CC are better suited for applications such as movie selection (see Sect. 1) rather than, say, parliamentary elections. Indeed, these rules sacrifice numerical proportionality of the representation in favor of committee diversity, by aiming to represent as many different views as possible (including views of very small minorities, to the extent that this is possible for the given committee size). In consequence, these rules are good for recommendation systems, which simply need to present users with ‘committees’ of items so as to maximize the number of customers (voters) who are satisfied with at least one of the items on offer. As an extreme example, if $k{>}1$ and there is a single candidate ranked first by all voters, then both $\ell _1$-CC and Greedy-CC may output any committee that includes this candidate. In contrast, both $\ell _1$-Monroe and Greedy-Monroe can only assign this candidate to about $\frac{n}{k}$ voters and have to pick the rest of the committee so as to maximize the satisfaction of the remaining voters.

The following examples show that $\ell _1$-CC and $\ell _1$-Monroe differ from their greedy counterparts.

#### Example 1

Let $E = (C,V)$ be an election with $C = \{a,b,c,d\}$ and the following four votes:

$$\begin{aligned} v_1:&a\succ c\succ b\succ d, \qquad v_2:b\succ c\succ a\succ d,\\ v_3:&a\succ c\succ d\succ b, \qquad v_4:b\succ c\succ d\succ a. \end{aligned}$$

Let $k=2$. Candidate *c* is the unique Borda winner in this election, so Greedy-CC will choose him in the first round (and then *a* or *b* in the second round). In contrast, the unique winning committee under $\ell _1$-CC is $\{a, b\}$.

#### Example 2

Let $E = (C,V)$ be an election with $C = \{a,b,c,d,e,f\}$ and the following four votes:

$$\begin{aligned} v_1:&a\succ c\succ d \succ e \succ b \succ f, \qquad v_2:b\succ a\succ c \succ d \succ e \succ f, \\ v_3:&a\succ c\succ d \succ e \succ b \succ f, \qquad v_4:f\succ a\succ e\succ d \succ c \succ b. \end{aligned}$$

Let $k=2$. Greedy-Monroe assigns candidate *a* to $v_1$ and $v_3$ in the first round. It then selects *b* or *f* in the second round. In contrast, $\ell _1$-Monroe chooses $\{a, c\}$ (with *a* assigned to $v_2$ and $v_4$ and *c* assigned to $v_1$ and $v_3$, or *c* assigned to $v_1$ and $v_2$ and *a* assigned to $v_3$ and $v_4$).

**Computational complexity of winner determination ** For $\ell$-Monroe and $\ell$-CC with $\ell \in \{\ell _1, \ell _{\min }\}$, it is known that finding even a single winning committee is computationally hard (Procaccia et al. 2008; Lu and Boutilier 2011; Betzler et al. 2013). However, many variants of these rules are fixed-parameter tractable, can be efficiently approximated, or admit polynomial-time algorithms when voters’ preferences are drawn from restricted domains (Lu and Boutilier 2011; Betzler et al. 2013; Cornaz et al. 2012; Yu et al. 2013; Skowron et al. 2015, 2016; Skowron and Faliszewski 2015; Skowron et al. 2015; Elkind and Ismaili 2015; Peters and Elkind 2016). For other rules that we study, one can output *some* winning committee in polynomial time. Moreover, for SNTV, Bloc, and *k*-Borda it is easy to decide whether a given committee *W* of size *k* is among the winning committees for a given election, i.e., whether the ties that occur during the application of the rule can be broken so that the rule outputs *W*. In contrast, for STV the latter problem is NP-hard even for $k=1$ (Conitzer et al. 2009). For Greedy-CC and Greedy-Monroe the complexity of checking if a given committee may win under some way of breaking ties is currently unknown. However, both of these rules can be combined with a simple tie-breaking mechanism so that the resulting rule is polynomial-time computable; moreover, these mechanisms do not interfere with the axiomatic analysis presented in this work.

### Two types of multiwinner rules

Perhaps surprisingly, it turns out that many of the rules introduced so far have very similar internal structure. Below we present two natural ways of identifying these similarities.

**Best-** $\mathbf{k}$ **Rules** SNTV and *k*-Borda are natural extensions of Plurality and Borda to the multiwinner setting: we sort the candidates in the order of decreasing scores (breaking ties in all possible ways) and pick the top *k* ones.

We remind the reader that a *social preference function* is a mapping that, given an election (*C*, *V*), returns a set of tied linear orders over *C*; a *social welfare function*, given an election (*C*, *V*), returns a single (possibly weak) order over *C*. By considering all linear orders that refine this weak order (which can be seen as a variant of the parallel-universes tie-breaking), we can convert a social welfare function into a social preference function; this enables us to treat social welfare functions as special cases of social preference functions. Hence a social preference function is a more general object than a social welfare function.

#### Definition 1

A committee selection rule ${{\mathcal {R}}}$ is a *best-k rule* if there exists a social preference function *F* such that for each election $E = (C, V)$ with $\Vert C\Vert =m$ and each $k \in [m]$ the set ${{\mathcal {R}}}(E, k)$ consists of all sets *W* such that there is an order $\mathord {\succ }$ in *F*(*E*) that ranks the candidates in *W* in the top *k* positions (i.e., $\Vert W\Vert =k$ and $c \succ d$ for each $c \in W$ and $d \in C {\setminus } W$).

SNTV and *k*-Borda are best-*k* rules. Indeed, every score vector $\mathbf{s}=(s_1, \ldots , s_m)$, where $s_1\ge s_2\ge \cdots \ge s_m$, induces a best-*k* rule. Specifically, any such score vector defines a social welfare function $F_\mathbf{s}$ that ranks all alternatives according to their $\mathbf{s}$-scores; we say that $F_\mathbf{s}$ is the *scoring social welfare function associated with the score vector* $\mathbf{s}$. We can then convert $F_\mathbf{s}$ into a social preference function as described above, and consider the associated committee selection rule. As we will see later, perhaps unexpectedly, Bloc is not a best-*k* rule.

We can also define a best-*k* rule based on the social preference function known as the Kemeny ranking (Kemeny 1959), and, somewhat surprisingly, we will later demonstrate that Greedy-CC is also a best-*k* rule. Thus, best-*k* rules are a more diverse group than one might at first expect.

**Committee scoring rules** Both *k*-Borda and $\ell _1$-CC can be viewed as generalizations of the Borda rule to the multiwinner case. To formalize this intuition, we will now define *committee scoring rules*. These rules can be seen as analogues of single-winner scoring rules, and include *k*-Borda, $\ell _1$-CC, and many other rules. We believe that identifying this class of rules is an important conceptual contribution of this paper.

Consider an election $E = (C,V)$ with *m* candidates, and a positive integer $k\in [m]$. Given a committee *S* with $\Vert S\Vert =k$ and a voter *v*, we define the *position of* *S* *in* *v* (denoted ${{{\mathrm {pos}}}}_v(S)$) to be the vector $(i_1, \ldots , i_k)$ that results from sorting the set $\{{{{\mathrm {pos}}}}_v(c) \mid c \in S\}$ in increasing order. We write $[m]_k$ to denote the set of all increasing length-*k* sequences of numbers from [*m*]; we interpret $[m]_k$ as the set of all possible committee positions for elections with *m* candidates and committee size *k*. For two committee positions from $[m]_k$, $I = (i_1, \ldots , i_k)$ and $J = (j_1, \ldots , j_k)$, we say that *I* *dominates* *J* (denoted $I \succeq J$) if and only if $i_\ell \le j_\ell$ for all $\ell \in [k]$.

An (*m*, *k*)-*committee scoring function* is a mapping $f_{m,k} :[m]_k \rightarrow {\mathbb {N}}$ such that for every pair of committee positions $I, J \in [m]_k$ with $I \succeq J$ it holds that $f(I) \ge f(J)$. The latter condition is a basic monotonicity requirement: it says that if *I* is obviously “not worse” than *J*, then the score associated with *I* is at least as high as that associated with *J*.

#### Definition 2

A *committee scoring function* *f* is a collection of (*m*, *k*)-committee scoring functions for all $m\in {{\mathbb {N}}}$ and all $k\in [m]$: $f = \{f_{m, k}\}_{m\in {{\mathbb {N}}}, k\in [m]}$. The *score* of a committee *S* of size *k* in an election $E = (C,V)$ with $\Vert C\Vert =m$ with respect to a committee scoring function $f = \{f_{m, k}\}_{m\in {{\mathbb {N}}}, k\in [m]}$ is defined as

$$\begin{aligned} \text {sc}_{f, E}(S)=\sum _{v \in V}f_{m, k}({{{\mathrm {pos}}}}_v(S)). \end{aligned}$$

A *committee scoring rule* $\mathcal{R}_f$ *associated with f* is a committee selection rule that, given an election *E* and a target committee size *k*, outputs all size-*k* committees that have the maximum score with respect to *f*.

Many of the rules that we study are, in fact, committee scoring rules.

#### Example 3

Let *m* be the number of candidates and let *k* be the target committee size. For each $\ell \in [m]$ define $\alpha _\ell :[m] \rightarrow \{0, 1\}$ by setting $\alpha _\ell (i) = 1$ if $i \le \ell$ and $\alpha _\ell (i) = 0$ otherwise. For each $i\in [m]$, define $\beta (i) = m-i$. SNTV, Bloc, *k*-Borda, and $\ell _1$-CC are committee scoring rules defined by the following committee scoring functions:

$$\begin{aligned} f_{m, k}^{{{\mathrm {SNTV}}}}(i_1, \ldots , i_k)&= \alpha _1(i_1), \\ f_{m, k}^{{{\mathrm {Bloc}}}}(i_1, \ldots , i_k)&= \textstyle \sum \limits _{t=1}^{k}\alpha _k(i_t), \\ f_{m, k}^{{{k\hbox {-}\mathrm {Borda}}}}(i_1, \ldots , i_k)&= \textstyle \sum \limits _{t=1}^k\beta (i_t), \\ f_{m, k}^{{{\mathrm {CC}}}}(i_1, \ldots , i_k)&= \beta (i_1). \end{aligned}$$

Example 3 motivates the following definition.

#### Definition 3

A committee scoring function $f = \{f_{m, k}\}_{m\in {{\mathbb {N}}}, k\in [m]}$ is said to be *separable* if it can be written as

1 $$\begin{aligned} f_{m, k}(i_1, \ldots , i_k)=\gamma ^m(i_1)+\cdots +\gamma ^m(i_k), \end{aligned}$$

for some family of functions $\gamma = \{\gamma ^m\}_{m \in {\mathbb {N}}}$, where each $\gamma ^m$ is a non-increasing function from [*m*] to ${\mathbb {N}}$ that does not depend on *k* and satisfies $\gamma ^m(1)>\gamma ^m(m)$. A committee scoring rule ${{\mathcal {R}}}$ is said to be *separable* if there exists a separable committee scoring function *f* such that ${{\mathcal {R}}}={{\mathcal {R}}}_f$.

It is immediate that $f^{{{k\hbox {-}\mathrm {Borda}}}}$ is separable. To see that $f^{{{\mathrm {SNTV}}}}$ is separable as well, note that we have $\alpha _1(i_1) = \alpha _1(i_1)+\alpha _1(i_2)+\cdots +\alpha _1(i_k)$, because $i_2, \ldots , i_k>1$ and $\alpha _1(i)=0$ for $i>1$. On the other hand, $f^{{{\mathrm {CC}}}}$ and $f^{{{\mathrm {Bloc}}}}$ are not separable (in particular, $f^{{{\mathrm {Bloc}}}}$ does not have this property because $\alpha _k$ depends on *k*).

Consider a separable committee scoring rule $\mathcal{R}_f$ with a scoring function *f* of the form (1), and set $s_i = \gamma ^m(i)$ for $i\in [m]$. Since $\gamma ^m$ is non-increasing and satisfies $\gamma ^m(1)>\gamma ^m(m)$, we have $s_1\ge s_2\ge \cdots \ge s_m$, $s_1>s_m$, and therefore ${\mathbf {s}} = (s_1, \ldots , s_m)$ is a valid score vector. Now, the total score of a committee $S=\{a_1, \ldots , a_k\}$ with respect to *f* in an election $E = (C, V)$ with $V = (v_1, \ldots , v_n)$ is

2 $$\begin{aligned} \text {sc}_{f, E}(S) = \sum _{i=1}^n\sum _{j=1}^k \gamma ^m(\text {pos}_{v_i}(a_j)) = \sum _{i=1}^n\sum _{j=1}^k s_{\text {pos}_{v_i}(a_j)}=\sum _{i=1}^k \text {sc}_\mathbf{s}(a_i). \end{aligned}$$

Hence, $\mathcal{R}_f$ is the best-*k* rule induced by the scoring social welfare function associated with the score vector $\mathbf{s}$. That is, we have the following theorem.

#### Theorem 1

Every separable committee scoring rule is a best-*k* rule for some scoring social welfare function.

It can be shown that Bloc is not a best-*k* rule (this will follow, in particular, from Theorem 2 and Proposition 3) and hence it is not a separable committee scoring rule. Indeed, in some ways Bloc is quite different from separable rules (for instance, it satisfies the fixed majority property, defined in the end of Sect. 4, which is failed by many separable rules). On the other hand, at the formal level Bloc does look very similar to separable committee scoring rules, and, indeed, it shares a number of common features with them. The reason is that both separable committee scoring rules and Bloc belong to the class of weakly separable committee scoring rules, which we define below.

#### Definition 4

A committee scoring rule ${{\mathcal {R}}}_f$ associated with a committee scoring function $f=\{f_{m, k}\}_{m\in {{\mathbb {N}}}, k\in [m]}$ is said to be *weakly separable* if there exists a family of non-increasing functions $\gamma ^m_k:[m] \rightarrow {\mathbb N}$, $m\in {{\mathbb {N}}}, k\in [m]$, such that for each $m \in {\mathbb N}$ and each $k \in [m]$ it holds that $\gamma ^m_k(1)>\gamma ^m_k(m)$ and for every sequence $0< i_1< \cdots < i_k \le m$ we have

$$\begin{aligned} f_{m, k}(i_1, \ldots , i_k) = \sum _{t=1}^k\gamma _k^m(i_t). \end{aligned}$$

We emphasize that, unlike a separable committee scoring rule, a weakly separable committee scoring rule may be based on a scoring social welfare function whose score vector depends on *k* (as in the case of Bloc).

Our next proposition shows that weakly separable committee scoring rules are attractive rules from an algorithmic perspective; moreover, in Sect. 7 we show that they have an interesting monotonicity property.

#### Proposition 1

If ${{\mathcal {R}}}$ is a weakly separable committee scoring rule that corresponds to a family of functions $\{\gamma ^m_k \mid m\in {{\mathbb {N}}}, k\in [m]\}$, and each function $\gamma ^m_k$ is polynomial-time computable, then ${{\mathcal {R}}}$ itself is polynomial-time computable.

On the other extreme, we have committee scoring rules whose committee scoring functions $f_{m, k}(i_1, \ldots , i_k)$ depend solely on $i_1$. We refer to such rules as *representation-focused rules*. Indeed, if $f_{m, k}$ only depends on $i_1$, a voter evaluating a committee only takes in consideration the best member of that committee (her representative). We note that representation-focused rules are exactly the $\ell _1$-$\alpha$-CC rules, i.e., the variants of the utilitarian Chamberlin–Courant rule that may use satisfaction functions other than the Borda satisfaction function.

## Axioms

We now present some properties (axioms) that may be desirable for committee selection rules. We use the standard axioms for single-winner rules as our starting point, and modify them on the basis of ideas from the literature that are specific to committee selection. Due to our choice of focus, we do not include properties based on the Condorcet principle, such as, e.g., stability, as defined by Gehrlein (1985). We stress that, since committee selection rules have a very diverse range of applications, our properties should not necessarily be understood in the normative way: the desirability of a particular property can only be evaluated in the context of a specific application. Throughout this section, we write ${{\mathcal {R}}}$ to denote a multiwinner rule; given an election $E=(C, V)$ and a positive integer *k* with $k\le \Vert C\Vert$, ${{\mathcal {R}}}(E, k)$ outputs a non-empty set of size-*k* subsets of *C*.

Our first axiom is *nonimposition*. It requires that each size-*k* set of candidates can be the unique winner. This is a basic requirement that is trivially satisfied by all rules that we consider.

- **Nonimposition** For each set of candidates *C* and each *k*-element subset *W* of *C*, there is an election $E = (C,V)$ such that ${{\mathcal {R}}}(E,k) = \{W\}$.

The next three axioms—consistency, homogeneity, and monotonicity—are adapted from the single-winner setting. For the first two, the adaptation is straightforward.

- **Consistency** For every pair of elections $E_1 = (C,V_1)$, $E_2 = (C,V_2)$ over a candidate set *C* and each $k \in [\Vert C\Vert ]$, if ${{\mathcal {R}}}(E_1,k) \cap {{\mathcal {R}}}(E_2,k) \ne \emptyset$ then ${{\mathcal {R}}}(E_1+E_2,k) = {{\mathcal {R}}}(E_1,k) \cap {{\mathcal {R}}}(E_2,k)$.
- **Homogeneity** For every election $E = (C,V)$, each $k \in [\Vert C\Vert ]$, and each $t \in {{\mathbb {N}}}$ it holds that ${{\mathcal {R}}}(tE,k) = {{\mathcal {R}}}(E,k)$.

We now consider monotonicity. If *c* belongs to a winning committee *W* then, generally speaking, we cannot expect *W* to remain winning when *c* is moved forward in some vote. For example, this shift may hurt other members of *W*. Indeed, none of our rules satisfies this strict version of monotonicity. However, there are two natural relaxations of this condition. One option is to require that after the shift *c* belongs to *some* winning committee. Alternatively, we may restrict forward movements of *c*, prohibiting it to overtake other members of *W*. (We point the reader to the work of Sanver and Zwicker (2012) for an extensive discussion of monotonicity in the context of irresolute voting rules.)

- **Monotonicity** For every election $E = (C,V)$, each $c \in C$, and each $k \in [\Vert C\Vert ]$, if $c\in W$ for some $W \in {{\mathcal {R}}}(E,k)$, then for every $E'$ obtained from *E* by shifting *c* one position forward in some vote *v* it holds that:

1. for *candidate monotonicity*: $c\in W'$ for some $W' \in {{\mathcal {R}}}(E',k)$, and
2. for *non-crossing monotonicity*: if *c* was ranked immediately below some $b\not \in W$, then $W \in {{\mathcal {R}}}(E',k)$.

Our next axiom, *committee monotonicity*, is specific to committee selection, as it deals with changing the size of the desired committee. Intuitively, it requires that when we increase the target committee size, none of the already selected candidates should be dropped. Our phrasing is somewhat involved because ${{\mathcal {R}}}$ may return a set of tied committees; a similar notion for resolute multiwinner rules, i.e., rules that always output a single committee, was introduced earlier by Barberà and Coelho (2008) under the name of *enlargement consistency*.

- **Committee monotonicity** For every election $E = (C,V)$ the following conditions hold:

1. for each $k \in [\Vert C\Vert -1]$, if $W \in {{\mathcal {R}}}(E,k)$ then there exists a $W' \in {{\mathcal {R}}}(E,k+1)$ such that $W \subseteq W'$;
2. for each $k \in [\Vert C\Vert -1]$, if $W \in {{\mathcal {R}}}(E,k+1)$ then there exists a $W' \in {{\mathcal {R}}}(E,k)$ such that $W' \subseteq W$.

The second condition in the definition above aims to prevent the following situation. Consider an election *E* with candidate set $C = \{a,b,c, \ldots \}$ and a rule ${{\mathcal {R}}}$ with ${{\mathcal {R}}}(E,1) = \{\{a\}\}$, ${{\mathcal {R}}}(E,2) = \{\{a,b\}, \{b,c\}\}$. Intuitively, ${{\mathcal {R}}}$ is not committee-monotone because of the unexpected appearance of the committee $\{b, c\}$ in ${{\mathcal {R}}}(E,2)$, but this is not captured by condition (1) above. Under our definition, ${{\mathcal {R}}}$ is not committee-monotone because it violates condition (2).

In Sect. 5 we discuss committee monotonicity in detail and use it to axiomatize best-*k* rules. We note that committee monotonicity in the context of multiwinner voting has been discussed by other authors, but under different names and usually by arguing that it is paradoxical that a given voting rule fails committee monotonicity (Staring 1986; Ratliff 2003; Barberà and Coelho 2008). In contrast, we argue (Sect. 5) that in some applications failing this axiom may be seen as a desirable feature of a committee selection rule.

The next three axioms represent three implementations of Dummett’s condition known as *proportionality for solid coalitions* (Dummett 1984). Dummett’s original proposal is as follows: Consider an election with *n* voters where the goal is to pick *k* candidates. If for some $\ell \in [k]$ there is a group of $\frac{\ell n}{k}$ voters that all rank the same $\ell$ candidates on top, these $\ell$ candidates should be in the (unique) winning committee. This requirement, which aims to capture the idea of proportional representation, seems to be very strong, and very few rules satisfy it.^3^ The following three axioms are weaker, but reflect the same idea (for a discussion of similar ideas in the context of approval-based rules, we point the readers to the work of Aziz et al. (2017) on *justified representation*).

- **Solid coalitions** For every election $E = (C,V)$ and each $k \in [\Vert C\Vert ]$, if at least $\frac{\Vert V\Vert }{k}$ voters rank some candidate *c* first then *c* belongs to every committee in ${{\mathcal {R}}}(E,k)$.
- **Consensus committee** For every election $E = (C,V)$ and each $k \in [\Vert C\Vert ]$, if there is a *k*-element set *W*, $W \subseteq C$, such that each voter ranks some member of *W* first and each member of *W* is ranked first by either $\lfloor \frac{\Vert V\Vert }{k} \rfloor$ or $\lceil \frac{\Vert V\Vert }{k} \rceil$ voters then ${{\mathcal {R}}}(E,k) = \{W\}$.
- **Unanimity** For every election $E = (C,V)$ and each $k \in [\Vert C\Vert ]$, if each voter ranks the same *k* candidates *W* on top (possibly in different order), then ${{\mathcal {R}}}(E,k) = \{W\}$ (strong unanimity) or $W \in {{\mathcal {R}}}(E,k)$ (weak unanimity).

We emphasize that the axioms in our list are suitable for rules that are preference-based and, moreover, are—in some broad sense—close to scoring rules. There are, however, axioms for preference-based rules that are geared towards the Condorcet principle. The following axiom is an example (Debord 1993), though it can also be seen as a generalization of the unanimity property.

- **Fixed majority** For every election $E = (C,V)$ and each $k \in [\Vert C\Vert ]$, if there is a *k*-element set *W*, $W \subseteq C$, such that a strict majority of voters rank all members of *W* above all non-members of *W*, then ${{\mathcal {R}}}(E,k) = \{W\}$.

Almost all rules considered in this paper fail to satisfy this axiom. For most of them, this is already the case in the single-winner setting, i.e., when $k=1$. Indeed, Plurality is the only single-winner scoring rule guaranteeing that a candidate ranked on top by a majority of the voters is the unique winner. However, quite interestingly, Bloc does satisfy the fixed majority property. To see this, consider an election (*C*, *V*) with $\Vert V\Vert =n$ and a committee *W* such that a majority of voters rank all members of *W* in top *k* positions. Under Bloc each candidate in *W* gets at least $\lfloor n/2\rfloor +1$ points, whereas each candidate in $C{\setminus } W$ gets at most $\lfloor n/2\rfloor$ points, which means that the Bloc score of every committee $W'\ne W$ is lower than that of *W*. This is yet another feature that distinguishes Bloc from separable committee scoring rules (even though Bloc is weakly separable). For more detailed discussion of fixed-majority consistency in the context of committee scoring rules, we point the readers to the follow-up work of Faliszewski et al. (2016b).

In the remainder of the paper, we consider the axioms listed in this section one by one, and, for each of them, we determine which of the voting rules described in Sect. 3 satisfy it. A summary of our results is provided in Table 1 in Sect. 10.

## Committee monotonicity

Some authors believe that committee monotonicity is an important requirement. In particular, Staring (1986) demonstrated that the Bloc rule is very far from being committee monotonic, and called this phenomenon the *increasing-committee-size paradox*. Specifically, he described a profile where the winning committee of size three was disjoint from the winning committee of size two, and the winning committee of size four was disjoint from both of them. A variant of the increasing-committee-size paradox has been recently considered by Kamwa and Merlin (2015). For several other rules (not considered in our paper), similar phenomena were observed by Ratliff (2003), in a paper titled “Some Startling Inconsistencies when Electing Committees”. Barberà and Coelho (2008) argue that for shortlisting it is desirable to consider weakly Condorcet-consistent rules. They then show that weak Condorcet-consistency (which, in this context, they call *stability*) and committee monotonicity are incompatible, and argue that this is one reason why (weakly) Condorcet-consistent rules are not used in practice.

In contrast, we believe that the desirability of committee monotonicity depends strongly on the application. In particular, we agree with Barberà and Coelho (2008) that if we are choosing finalists of a competition, then it is imperative to use a rule that has this property; however, in the context of proportional representation insisting on a committee-monotone rule may prevent us from selecting a truly representative committee. Indeed, the latter point was already noted by Black. In his classical work Black (1958) considers a society with single-peaked preferences regarding the left-right political spectrum. He observes that if we are choosing a single candidate (i.e., if $k=1$) then it is most natural to select a candidate that holds the most centrist position (i.e., the top choice of the median voter). However, if we are to select two candidates to represent the society (i.e., if $k=2$), then, intuitively, a committee consisting of a “moderate left-wing” candidate and a “moderate right-wing” candidate would be more representative than any committee of the form $\{c, d\}$, where *c* is the top choice of the median voter: if *d* is to the left of *c*, the right-wing voters are neglected, and if *d* is to the right of *c*, the left-wing voters are neglected.

The above intuition is further strengthened by the fact that committee monotonicity axiomatically characterizes the class of best-*k* rules.

### Theorem 2

A committee selection rule is committee-monotone if and only if it is a best-*k* rule.

### Proof

Let ${{\mathcal {R}}}$ be a best-*k* rule and let *F* be the associated social preference function. Consider an election $E = (C,V)$. Pick $k\in [\Vert C\Vert -1]$ and $W\in {{\mathcal {R}}}(E,k)$. Since ${{\mathcal {R}}}$ is a best-*k* rule, there is an order $\mathord {\succ }$ in *F*(*E*) that ranks members of *W* in top *k* positions. Let $w'$ be the candidate ranked in position $k+1$ in $\mathord {\succ }$. Then $W \cup \{w'\}$ is in ${{\mathcal {R}}}(E,k+1)$. A similar argument shows that ${{\mathcal {R}}}$ satisfies the second committee-monotonicity condition.

Conversely, assume that ${{\mathcal {R}}}$ satisfies committee monotonicity. We will show that it is a best-*k* rule by identifying the underlying social preference function *F*. Consider an election $E = (C,V)$ with $C = \{c_1, \ldots , c_m\}$. Let *F*(*E*) be the set of all linear orders $\mathord {\succ }$ over *C* that satisfy the following condition: If $c_{\pi (1)} \succ c_{\pi (2)} \succ \cdots \succ c_{\pi (m)}$ for some permutation $\pi$ of [*m*] then there is a sequence of committees $W_1, \ldots , W_m$ such that $W_i = \{c_{\pi (1)}, \ldots , c_{\pi (i)}\}$ and $W_i \in {{\mathcal {R}}}(E,i)$ for $i\in [m]$. The two conditions from the definition of committee monotonicity imply that *F* indeed defines ${{\mathcal {R}}}$. $\square$

By combining Theorems 1 and 2, we conclude that all separable committee scoring rules, including, in particular, SNTV and *k*-Borda, satisfy committee monotonicity. We will now show that Greedy-CC satisfies committee monotonicity as well, and hence it is a best-*k* rule.

### Proposition 2

Greedy-CC satisfies committee monotonicity.

### Proof

Given an election $$E=(C, V)$$ and an integer *k*, Greedy-CC performs *k* iterations, choosing one member of the committee at each step. Importantly, the member of the committee picked in each iteration depends only on which members were chosen previously and does not depend on *k*. Thus, each sequence of $$\Vert C\Vert $$ choices defines a linear order on *C*. Let $$F_ CC (E)$$ be the set of all linear orders that can be obtained in this way (for all ways of breaking the intermediate ties). By construction, the multiwinner rule associated with $$F_ CC $$ is exactly Greedy-CC. $$\square $$

Theorem 1 shows that separable committee scoring rules are associated with scoring social welfare functions. In contrast, Greedy-CC cannot be defined in such a way and, in particular, the social preference function $$F_ CC $$ constructed in the proof of Proposition 2 does not correspond to a social welfare function.

### Example 4

Consider an election $E=(C, V)$, where $C=\{a, b, c, d, e\}$ and *V* contains:

1. 4 voters with preference order $a\succ b\succ c \succ d \succ e$,
2. 4 voters with preference order $b\succ a\succ c \succ d \succ e$,
3. 1 voter with preference order $c\succ d\succ e \succ a \succ b$, and
4. 1 voter with preference order $c\succ d\succ e \succ b \succ a$.

One can verify that

$$\begin{aligned} F_ CC (E)&= \{ a\succ c \succ b \succ d \succ e, \ \ a \succ c \succ b \succ e \succ d, \\&\quad b\succ c \succ a \succ d \succ e, \ \ b \succ c \succ a \succ e \succ d\}. \end{aligned}$$

Suppose for the sake of contradiction that $$F_ CC $$ is obtained from a social welfare function $$F'$$. Since both *a* and *b* appear in top positions in linear orders in $$F_ CC (E)$$, $$F'(E)$$ is a weak order of the form $$\{a, b\}\succ \cdots $$. But then $$F_ CC (E)$$ would also have to contain a linear order of the form $$a\succ b \succ \cdots $$. Since it does not, we reached a contradiction. In fact, this example shows that Greedy-CC does not correspond to any social welfare function. Indeed, for election *E* and $$k=1$$ Greedy-CC outputs committees $$\{a\}$$ and $$\{b\}$$. Thus, if such a function existed, for $$k=2$$ Greedy-CC would have to output committee $$\{a,b\}$$, but it does not.

The remaining rules considered in our paper fail committee monotonicity (as mentioned above, the case of Bloc was resolved by Staring (1986); the proof of Proposition 3 includes an explicit construction for Bloc for the sake of completeness).

### Proposition 3

STV, Bloc, $\ell _1$-CC, $\ell _{\min }$-CC, $\ell _1$-Monroe, $\ell _{\min }$-Monroe, and Greedy-Monroe do not satisfy committee monotonicity.

### Proof

Let us first consider $\ell _1$-CC, $\ell _{\min }$-CC, $\ell _1$-Monroe, $\ell _{\min }$-Monroe, and Greedy-Monroe. Let $E = (C,V)$ be an election with $C = \{a,b,c,d\}$ and the following four votes:

$$\begin{aligned} v_1:&a\succ c\succ b\succ d, \quad v_2:b\succ c\succ a\succ d, \\ v_3:&a\succ c\succ d\succ b, \quad v_4:b\succ c\succ d\succ a. \end{aligned}$$

For $k=1$ the unique winning committee is $\{c\}$, but for $k=2$ the unique winning committee is $\{a,b\}$.

For Bloc, consider an election $E = (C,V)$ with $C = \{a,b,c\}$ and 4 votes:For $k=1$ the unique winning committee under Bloc is $\{a\}$, but for $k=2$ the unique winning committee under Bloc is $\{b,c\}$.

Our example for STV is somewhat more involved. Consider $E = (C,V)$ with $C = \{a,b,c,d\}$ and 24 voters. There are

1. 11 voters with preference order $a \succ b \succ c \succ d$,
2. 3 voters with preference order $b \succ c \succ a \succ d$,
3. 4 voters with preference order $c \succ d \succ a \succ b$, and
4. 6 voters with preference order $d \succ c \succ a \succ b$.

For $k=1$, the Droop quota is $\lfloor \frac{24}{2}\rfloor +1 = 13$. In the first round no candidate meets the quota, so STV eliminates the candidate with the lowest Plurality score, that is, *b*. In the next round still no candidate meets the quota, so STV eliminates *d*. In the resulting election *c* has Plurality score 13 and is the unique winner for $k=1$.

For $k=2$, the Droop quota is $\lfloor \frac{24}{3}\rfloor +1 = 9$. Thus in the first round STV picks *a* and removes it from the election together with 9 voters that rank it first. The remaining two voters that supported *a* transfer their votes to *b*, who now has Plurality score 5. In the next round no candidate meets the quota, so STV eliminates *c*. After that *d* has Plurality score 10 and is selected. The unique winning committee is $\{a,d\}$. $\square$

### Corollary 1

Bloc, $\ell _1$-CC, $\ell _{\min }$-CC, $\ell _1$-Monroe, $\ell _{\min }$-Monroe, Greedy-Monroe, and STV are not best-*k* rules.

## Dummett’s proportionality

Properties in the spirit of Dummett’s proportionality condition (with the exception of unanimity) are geared toward rules that aim to achieve proportional representation of the voters. Thus, the results of this section can be used to judge committee selection rules from this perspective.

We start by considering the solid coalitions property. It is easy to see that it is satisfied by SNTV, and also by STV when the number of voters is sufficiently large.

### Proposition 4

SNTV has the solid coalitions property. STV has the solid coalitions property whenever the number of voters *n* and the target committee size *k* satisfy $n \ge k(k+1)$.

### Proof

Consider first SNTV. Suppose that in an election $E=(C, V)$ with $\Vert V\Vert =n$ some candidate *c* is ranked first by at least $\frac{n}{k}$ voters. By a counting argument, there can be at most $k-1$ other candidates whose Plurality score is at least $\frac{n}{k}$. Thus, *c* is included in each winning committee.

For STV, the Droop quota *q* equals $\lfloor \frac{n}{k+1}\rfloor +1$; if $n \ge k(k+1)$, then $q\le \frac{n}{k}$. In this case, if there is a candidate *c* who is ranked first by at least $\frac{n}{k}$ voters then *c* will be included in each winning committee. $\square$

On the other hand, even though the solid coalitions property seems to be very much in spirit of the Chamberlin–Courant and Monroe rules, $\ell _1$-Monroe, $\ell _1$-CC, $\ell _{\min }$-Monroe, and $\ell _{\min }$-CC fail to satisfy it.

### Proposition 5

$\ell _1$-CC, $\ell _{\min }$-CC, $\ell _1$-Monroe, and $\ell _{\min }$-Monroe do not have the solid coalitions property.

### Proof

Consider an election with candidate set $C = \{a,b,c,d,e,f,g\}$ and 9 voters whose preference orders are

$$\begin{aligned} v_1 :&a \succ e \succ d \succ f \succ g \succ b \succ c,\\ v_2 :&b \succ f \succ d \succ e \succ g \succ a \succ c,\\ v_3 :&c \succ g \succ d \succ e \succ f \succ a \succ b,\\ v_4 :&a \succ e \succ d \succ f \succ g \succ b \succ c,\\ v_5 :&b \succ f \succ d \succ e \succ g \succ a \succ c,\\ v_6 :&c \succ g \succ d \succ e \succ f \succ a \succ b,\\ v_7 :&d \succ a \succ e \succ f \succ g \succ b \succ c,\\ v_8 :&d \succ b \succ e \succ f \succ g \succ a \succ c,\\ v_9 :&d \succ c \succ e \succ f \succ g \succ a \succ b. \end{aligned}$$

One can verify that for $k=3$ neither utilitarian nor egalitarian versions of the Chamberlin–Courant and Monroe rules elect a committee that contains *d*, even though this would be required by the solid coalitions property. Indeed, for the committee $\{a,b,c\}$ the total satisfaction is $6(\Vert C\Vert -1)+3(\Vert C\Vert -2) = 9\Vert C\Vert -12$ in the utilitarian version and $\Vert C\Vert -2$ in the egalitarian version. However, if *d* is in the committee, then at most two of the remaining candidates are also in the committee and so the total satisfaction is, respectively, at most $9\Vert C\Vert -13$ and $\Vert C\Vert -3$. $\square$

Interestingly, Greedy-Monroe performs better in this regard than $\ell _1$-Monroe.

### Proposition 6

Greedy-Monroe has the solid coalitions property.

### Proof

Consider an *n*-voter election where some candidate *c* is ranked first by at least $\frac{n}{k}$ voters (where *k* is the target committee size). Greedy-Monroe starts by choosing candidates ranked first by at least $\frac{n}{k}$ voters. By the time it considers *c*, each of the voters that rank *c* first remains unassigned, so it picks *c*. $\square$

We believe that the solid coalitions property is desirable, but not crucial for applications that aim at proportional representation (e.g., parliamentary elections): indeed, a voter may be reasonably well represented even if her top choice is not included in the committee. In contrast, the consensus committee property, which we discuss next, seems to be fundamental. Indeed, it is satisfied by almost all rules that aim to achieve proportional representation.

When *k* divides *n*, the consensus committee property is satisfied by every rule that has the solid coalitions property. In particular, it is satisfied by SNTV, STV (if there are sufficiently many voters) and Greedy-Monroe. It is also satisfied by $\ell _1$-CC, $\ell _{\min }$-CC, $\ell _1$-Monroe, and $\ell _{\min }$-Monroe: this is immediate from the definitions of these rules. Interestingly, however, Greedy-CC fails the consensus committee property. This reveals a major deficiency of this rule: when deciding whether to include some candidate *c* into the committee, it takes into account the preferences of the voters to whom *c* would not be assigned. This is very problematic for a rule that seeks to approximate $\ell _1$-CC. Observe, however, that it is exactly this feature of Greedy-CC that makes this rule committee monotone.

### Proposition 7

Bloc, *k*-Borda and Greedy-CC do not have the consensus committee property (nor the solid coalitions property).

### Proof

Consider an election with $C = \{a,b,c,d\}$ and two voters with preference orders $b \succ c \succ d \succ a$ and $a \succ c \succ d \succ b$. We seek a committee of size $k = 2$. Then the consensus committee is $\{a,b\}$, but each of our rules includes *c* in each winning committee and thus fails the consensus committee property. $\square$

For SNTV, $\ell _1$-CC, and *k*-Borda, the above results can also be seen as incarnations of the following two more general results regarding committee scoring rules.

### Theorem 3

Let ${{\mathcal {R}}}$ be a separable committee scoring rule, and let $f=\{f_{m, k}\}_{m\in {{\mathbb {N}}}, k\in [m]}$ be the associated committee scoring function with $f_{m, k}(i_1, \ldots , i_k) = \sum _{t=1}^k\gamma (t)$ for some non-increasing function $\gamma$ with $\gamma (1)>0$, $\gamma (j)\ge 0$ for all $j\in {{\mathbb {N}}}$. Then

1. if $\gamma (2)>0$, then ${{\mathcal {R}}}$ fails the consensus committee property for $k \ge \frac{\gamma (1)}{\gamma (2)}$;
2. if $\gamma (2)>0$, ${{\mathcal {R}}}$ satisfies the consensus committee property for $k < \frac{\gamma (1)}{\gamma (2)}$ when the number of voters is sufficiently large;
3. if $\gamma (2)=0$, ${{\mathcal {R}}}$ satisfies the consensus committee property for all values of *k*.

### Proof

By Theorem 1 for a given number of candidates $m> k$ the rule ${{\mathcal {R}}}$ is a best-*k* rule for the scoring vector $\mathbf{s}=(s_1, \ldots , s_m)$, where $s_i=\gamma (i)$ for $i\in [m]$.

Suppose first that $k \ge \frac{\gamma (1)}{\gamma (2)}$. For each $m>k$, we construct an election $E = (C,V)$ with *m* candidates and $k+1$ voters as follows. Let $C = \{c_1, \ldots , c_m\}$. Each $c_i$, $i\in [k-1]$, is ranked first by exactly one voter, $c_k$ is ranked first by two voters, the voters that do not rank $c_1$ first rank it last, and each voter ranks $c_{k+1}$ second. Aside from that, the voters’ preference orders are arbitrary. If ${{\mathcal {R}}}$ had the consensus committee property, then $\{c_1, \ldots , c_k\}$ would be the unique winning committee. However, observe that the $\mathbf{s}$-score of $c_1$ is $s_1$, the $\mathbf{s}$-score of $c_{k+1}$ is $(k+1)s_2$, so $0 < \gamma (1) \le k\gamma (2)$ implies that $c_{k+1}$ has a higher $\mathbf{s}$-score than $c_1$. Moreover, the $\mathbf{s}$-score of each of the candidates $c_2, \ldots , c_k$ is higher than or equal to that of $c_1$. Hence, $\{c_1, \ldots , c_k\}$ is not a winning committee. Thus, ${{\mathcal {R}}}$ does not have the consensus committee property.

The case $\gamma (2)=0$ is captured by our analysis of SNTV. Suppose now that $\gamma (2) > 0$ and $k< \frac{\gamma (1)}{\gamma (2)}$. Fix $n > \frac{k\gamma (1)}{\gamma (1)-k\gamma (2)}$; note that $\frac{1}{n} < \frac{1}{k}-\frac{\gamma (2)}{\gamma (1)}$. Consider an election $E = (C,V)$ with $\Vert V\Vert =n$ where there is a group of candidates *W*, $\Vert W\Vert =k$, such that each voter ranks some member of *W* first and each member of *W* is ranked first by either $\lfloor \frac{n}{k} \rfloor$ or $\lceil \frac{n}{k} \rceil$ voters. Then the $\mathbf{s}$-score of each candidate in *W* is at least $\lfloor \frac{n}{k}\rfloor s_1$. On the other hand, the $\mathbf{s}$-score of a candidate $x\notin W$ is at most $ns_2$. By our choice of *n* we have $\lfloor \frac{n}{k}\rfloor s_1\ge n(\frac{1}{k}-\frac{1}{n})s_1> ns_2$. Thus, *W* is the unique winning committee for such values of *n*, and the consensus committee property is satisfied. $\square$

### Proposition 8

$\ell _1$-$\alpha$-CC has the consensus committee property if and only if $\alpha (1) > \alpha (2)$.

### Proof

Consider an election $E = (C,V)$ with $\Vert V\Vert =n$ where there is a group of candidates *W*, $\Vert W\Vert =k$, such that each voter ranks some member of *W* first and each member of *W* is ranked first by either $\lfloor \frac{n}{k} \rfloor$ or $\lceil \frac{n}{k} \rceil$ voters. If $\alpha (1) > \alpha (2)$ then *W* is the unique winning committee. Conversely, suppose that $\alpha (1) = \alpha (2)$, and consider an election $E'$ with 2*k* candidates and *k* voters, where each voter ranks a distinct pair of candidates in the top two positions. We have $\Vert {{\mathcal {R}}}(E', k)\Vert \ge 2^k$, yet the consensus committee property prescribes that ${{\mathcal {R}}}(E', k)$ is a singleton. $\square$

Our final instantiation of Dummett’s proportionality for solid coalitions is the unanimity property. Every committee scoring rule satisfies its weak variant.

### Theorem 4

Every committee scoring rule ${{\mathcal {R}}}$ satisfies weak unanimity.

### Proof

Consider an election $E = (C,V)$ with $\Vert C\Vert =m$ where every voter ranks candidates from some set *W*, $\Vert W\Vert =k$, in top *k* positions. Let $f=\{f_{m, k}\}_{m\in {{\mathbb {N}}}, k\in [m]}$ be the committee scoring function associated with ${{\mathcal {R}}}$. By definition, for every voter *v* in *V* and every set of candidates *Q* with $\Vert Q\Vert =k$ we have $f_{m, k}({{{\mathrm {pos}}}}_v(W)) \ge f_{m, k}({{{\mathrm {pos}}}}_v(Q))$. Thus, $W\in {{\mathcal {R}}}(E, k)$. $\square$

It is immediate that $\ell _1$-Monroe, $\ell _{\min }$-Monroe, Greedy-Monroe, Bloc and *k*-Borda satisfy strong unanimity. In contrast, SNTV, $\ell _1$-CC, $\ell _{\min }$-CC, and Greedy-CC do not have this property: given an election where all voters have the same preference order, each of these rules outputs all committees that include the candidate ranked first by all voters. Finally, we note that STV satisfies strong unanimity. Indeed, if there is a set of candidates *W*, $\Vert W\Vert =k$, such that each of the *n* voters ranks the candidates from *W* in top *k* positions, then in every round of STV there is a candidate from *W* that is ranked first by at least $\lceil \frac{n}{k}\rceil \ge \lfloor \frac{n}{k+1}\rfloor +1$ voters.

## Monotonicity

Being monotonic is a natural and easily satisfiable condition for single-winner rules. Among the few examples of prominent non-monotonic single-winner rules are STV and the Dodgson rule (see, e.g., the work of Brandt 2009). In contrast, for multiwinner rules monotonicity is a rather demanding property. However, all committee scoring rules satisfy candidate monotonicity, and all weakly separable committee scoring rules satisfy non-crossing monotonicity.

### Theorem 5

Every committee scoring rule ${{\mathcal {R}}}$ satisfies candidate monotonicity.

### Proof

Let $f=\{f_{m, k}\}_{m\in {{\mathbb {N}}}, k\in [m]}$ be the committee scoring function associated with ${{\mathcal {R}}}$. Consider an election $E = (C,V)$ with $\Vert C\Vert =m$. Let *W* be a committee in ${{\mathcal {R}}}(E,k)$ and let *c* be a candidate in *W*. Consider a vote $v \in V$ where *c* is not ranked first, and replace it with a vote $v'$ obtained from *v* by shifting *c* one position forward. Denote the resulting election by $E'$. By construction, we have $f_{m, k}({{{\mathrm {pos}}}}_{v'}(W)) \ge f_{m, k}({{{\mathrm {pos}}}}_v(W))$. On the other hand, for every committee $S\subseteq C{\setminus }\{c\}$ we have $f_{m, k}({{{\mathrm {pos}}}}_{v'}(S)) \le f_{m, k}({{{\mathrm {pos}}}}_v(S))$. Since *W* was a winning committee for *E*, if $W'\ne W$ is a winning committee for $E'$ it has to be the case that $c\in W'$. $\square$

### Theorem 6

Every weakly separable committee scoring rule ${{\mathcal {R}}}$ satisfies non-crossing monotonicity.

### Proof

Consider an election $E = (C,V)$ with $\Vert C\Vert =m$. Let *W* be a committee in ${{\mathcal {R}}}(E, k)$ and let *c* be a candidate in *W*. Let $f=\{f_{m, k}\}_{m\in {{\mathbb {N}}}, k\in [m]}$ be a committee scoring function associated with ${{\mathcal {R}}}$, where $f_{m, k}(i_1, \ldots , i_k)=\sum _{t=1}^k \alpha _k^m(i_t)$. Consider a vote *v* where *c* is ranked in position *i* and some candidate $d\not \in W$ is ranked just above *c*. Swap *c* and *d* in *v*. After the swap, every committee that contains *c* and not *d* gains the same number of points, namely, $\alpha _k^m(i-1) - \alpha _k^m(i)\ge 0$; every committee that contains both *c* and *d* (or neither *c* nor *d*) maintains the same score, and every committee that contains *d* but not *c* loses $\alpha _k^m(i-1) - \alpha _k^m(i) \ge 0$ points. Thus *W* is a winning committee in the new election. $\square$

In contrast, one of the implications of our next result is that a committee scoring rule that is not weakly separable may fail non-crossing monotonicity. (Indeed, in a follow-up paper, Faliszewski et al. (2016a) have shown that among committee scoring rules, only weakly separable rules satisfy non-crossing monotonicity.)

### Proposition 9

$\ell _1$-CC, $\ell _1$-Monroe, Greedy-CC, and Greedy-Monroe fail non-crossing monotonicity.

### Proof

Consider an election with candidate set $C = \{a,b,c,d,x_1, \ldots , x_6\}$ and six voters with the following preference orders:

$$\begin{aligned} v_1:&a \succ x_1 \succ c \succ b \succ d \succ \cdots , \quad v_2:a \succ x_2 \succ d \succ b \succ c \succ \cdots , \\ v_3:&b \succ x_3 \succ a \succ c \succ d \succ \cdots , \quad v_4:b \succ x_4 \succ d \succ c \succ a \succ \cdots , \\ v_5:&c \succ x_5 \succ a \succ b \succ d \succ \cdots , \quad v_6:c \succ x_6 \succ d \succ b \succ a \succ \cdots . \end{aligned}$$

The reader can verify that if the target committee size is $k=2$, $\ell _1$-CC outputs three winning committees, namely, $\{a,b\}$, $\{a,c\}$, and $\{b,c\}$, each with satisfaction $6\Vert C\Vert -11$. Non-crossing monotonicity requires $\{a, c\}$ to remain winning after *c* is shifted forward in $v_1$ by one position. However, if that happens, the satisfaction of $\{a,c\}$ does not change, whereas the satisfaction of $\{b,c\}$ increases to $6\Vert C\Vert -10$. The same construction works for $\ell _1$-Monroe, Greedy-CC, and Greedy-Monroe. $\square$

While $\ell _{\min }$-CC satisfies one of our monotonicity properties, $\ell _{\min }$-Monroe fails both of them.

### Proposition 10

$\ell _{\min }$-CC satisfies candidate monotonicity, but $\ell _{\min }$-Monroe fails it. Both $\ell _{\min }$-CC and $\ell _{\min }$-Monroe fail non-crossing monotonicity.

### Proof

To see that $\ell _{\min }$-CC satisfies candidate monotonicity, consider a winning committee *W* and a candidate $c\in W$. Shifting *c* forward has the following effect. The aggregate satisfaction of every committee that includes *c* either increases by one, or stays the same. The aggregate satisfaction of every committee not containing *c* either stays the same or decreases by one. Thus *c* belongs to at least one winning committee.

To show that $\ell _{\min }$-Monroe fails candidate monotonicity, we construct an election with candidate set $C = \{a,b,c,d\}$ and six voters with the following preference orders:

3 $$\begin{aligned} v_1:&a \succ c \succ d \succ b, \quad v_2:c \succ a \succ d \succ b, \nonumber \\ v_3:&b \succ d \succ c \succ a, \quad v_4:d \succ b \succ a \succ c, \nonumber \\ v_5:&d \succ a \succ c \succ b, \quad v_6:b \succ c \succ d \succ a . \end{aligned}$$

For $k=2$, the winning committees are $\{a,b\}$ and $\{c,d\}$, both with satisfaction $\Vert C\Vert -2$. If we shift *a* forward by one position in $v_4$, the satisfaction of $\{a,b\}$ decreases to $\Vert C\Vert -3$ but the satisfaction of $\{c,d\}$ does not change.

To see that $\ell _{\min }$-CC fails non-crossing monotonicity, consider an election with candidate set $C = \{a,b,c,x_1, \ldots ,x_{11}\}$ and six voters with the following preference orders:

$$\begin{aligned} v_1:&a \succ x_1 \succ x_2 \succ b \succ \cdots , \quad v_2:x_3 \succ x_4 \succ x_5 \succ c \succ \cdots , \\ v_3:&b \succ a \succ c \succ x_6 \succ \cdots , \,\,\quad v_4:b \succ a \succ c \succ x_7 \succ \cdots , \\ v_5:&b \succ c \succ x_8 \succ x_9 \succ \cdots , \quad v_6:a \succ c \succ x_{10} \succ x_{11} \succ \cdots . \end{aligned}$$

For $k=2$, the winning committees are $\{a, c\}$, $\{b,c\}$, $\{x_1,c\}$, and $\{x_2,c\}$, all with aggregate satisfaction $\Vert C\Vert -4$. Non-crossing monotonicity requires that if we shift *c* forward in $v_2$, the committee $\{b,c\}$ should still be winning. However, this committee’s satisfaction stays the same, whereas the satisfaction of $\{a,c\}$ increases to $\Vert C\Vert -3$. The same construction works for $\ell _{\min }$-Monroe. $\square$

The remaining committee selection rules studied in this paper fail each of our monotonicity criteria. For STV this is well-known even for $k=1$. The other three rules are captured by the following proposition.

### Proposition 11

$\ell _1$-Monroe, Greedy-Monroe, and Greedy-CC fail candidate monotonicity.

### Proof

For $\ell _1$-Monroe, we reuse the first construction in the proof of Proposition 10, i.e., we start with election (3) and move *a* forward in vote $v_4$.

For Greedy-Monroe, we construct an election with candidate set $C = \{a,b,c,d\}$ and 8 voters with the following preference orders:

$$\begin{aligned} v_1:&b \succ c \succ d \succ a \succ e, \quad v_2:d \succ c \succ b \succ a \succ e, \\ v_3:&a \succ e \succ d \succ b \succ c, \quad v_4:a \succ b \succ d \succ c \succ e, \\ v_5:&a \succ e \succ d \succ b \succ c, \quad v_6:b \succ d \succ a \succ c \succ e, \\ v_7:&d \succ c \succ b \succ a \succ e, \quad v_8:c \succ b \succ d \succ a \succ e. \end{aligned}$$

For $k = 2$, Greedy-Monroe outputs $\{a,c\}$ and $\{b,d\}$. Indeed, in the first iteration Greedy-Monroe picks either *a* or *b*. If it picks *a*, then in the second iteration it picks *c*. If it picks *b*, then in the second iteration it picks *d*. However, if we shift *c* forward by one position in $v_6$, only $\{b,d\}$ remains winning, as the algorithm can no longer pick *a* in the first iteration. Hence, Greedy-Monroe fails candidate monotonicity.

For Greedy-CC, we construct an election with candidate set $C = \{a,b,c,d\}$ and 6 voters with the following preference orders:

$$\begin{aligned} v_1:&a \succ b \succ c \succ d, \quad v_2:b \succ c \succ d \succ a,\\ v_3:&a \succ b \succ c \succ d, \quad v_4:c \succ b \succ d \succ a,\\ v_5:&a \succ b \succ c \succ d, \quad v_6:d \succ a \succ c \succ b. \end{aligned}$$

For $k=2$, Greedy-CC outputs $\{a,b\}$ and $\{a,c\}$. Indeed, in the first iteration it picks either *a* or *b*. If it picks *a* then in the second iteration it picks either *b* or *c*. If it picks *b* in the first iteration, in the second iteration it picks *a*. However, if we shift *c* forward by one position in $v_6$, then $\{a,b\}$ is the unique winning committee: in this case, in the first iteration Greedy-CC has to pick *b*, and then in the second iteration it picks *a*. Thus, Greedy-CC fails candidate monotonicity as well. $\square$

## Consistency and homogeneity

For single-winner rules, Young’s famous theorem (Young 1975) says that only scoring rules and their compositions satisfy consistency. The situation for multiwinner voting rules seems to be similar. Here, we show that every committee scoring rule satisfies consistency, whereas other rules we consider fail it. In a very recent paper, Skowron et al. (2016b) provide a Young-style characterization of committee scoring rules, which explains our observations.

### Theorem 7

Every committee scoring rule satisfies consistency.

### Proof

Let ${{\mathcal {R}}}$ be a committee scoring rule, and let *f* be the associated committee scoring function. Consider two elections $E_1=(C, V_1)$ and $E_2=(C, V_2)$ over a candidate set *C* such that ${{\mathcal {R}}}(E_1,k) \cap {{\mathcal {R}}}(E_2,k) \ne \emptyset$, and let *W* be some committee in ${{\mathcal {R}}}(E_1,k) \cap {{\mathcal {R}}}(E_2,k)$.

Consider an arbitrary committee *Q* of size *k*. Since $W\in {{\mathcal {R}}}(E_1,k) \cap {{\mathcal {R}}}(E_2,k)$,

$$\begin{aligned} \text {sc}_{f, E_1}(W)\ge \text {sc}_{f, E_1}(Q), \quad \text {sc}_{f, E_2}(W)\ge \text {sc}_{f, E_2}(Q). \end{aligned}$$

As $\text {sc}_{f, E_1+E_2}(S) = \text {sc}_{f, E_1}(S)+\text {sc}_{f, E_2}(S)$ for $S\in \{Q, W\}$, we obtain $\text {sc}_{f, E_1+E_2}(W)\ge \text {sc}_{f, E_1+E_2}(Q)$, i.e., $W\in {{\mathcal {R}}}(E_1+E_2, k)$.

Conversely, let *T* be some committee in ${{\mathcal {R}}}(E_1+E_2, k)$. Since *W* is a winning committee in both $E_1$ and $E_2$, we have $\text {sc}_{f, E_1}(W)\ge \text {sc}_{f, E_1}(T), \text {sc}_{f, E_2}(W)\ge \text {sc}_{f, E_2}(T)$. On the other hand, since $T\in {{\mathcal {R}}}(E_1+E_2, k)$, we have $\text {sc}_{f, E_1+E_2}(T) = \text {sc}_{f, E_1+E_2}(W)$. Thus, both of the inequalities above hold with equality, and hence $T\in {{\mathcal {R}}}(E_1, k)$, $T\in {{\mathcal {R}}}(E_2, k)$. $\square$

Now let us turn our attention to the negative results. Note first that some of the rules we consider cannot be consistent by Young’s theorem, because for $k=1$ they are not scoring rules (or their compositions). Specifically, this is the case for STV, $\ell _{\min }$-CC and $\ell _{\min }$-Monroe.

### Corollary 2

None of STV, $\ell _{\min }$-CC and $\ell _{\min }$-Monroe is consistent.

This argument does not apply to $\ell _1$-Monroe, Greedy-CC and Greedy-Monroe: for $k=1$ each of these rules is equivalent to Borda, which is consistent. For these rules, we provide a direct proof.

### Proposition 12

None of $\ell _1$-Monroe, Greedy-CC and Greedy-Monroe is consistent.

### Proof

For $\ell _1$-Monroe we consider two elections $E_1 = (C,V_1)$ and $E_2 = (C,V_2)$ over the candidate set $C = \{a,b,c,d,e\}$, where $V_1 = (v_1, v_2,v_3, v_4)$, $V_2 = (v_5, v_6,v_7, v_8)$, and the voters have the following preferences:Under $\ell _1$-Monroe, for $k=2$ committee $\{a,c\}$ wins in both $E_1$ and $E_2$, but in $E_1+E_2$ it has lower satisfaction than $\{a,b\}$.

For Greedy-CC we consider two elections $E_1 = (C,V_1)$ and $E_2 = (C,V_2)$ over the candidate set $C = \{a,b,c,d\}$, where $V_1 = (v_1, v_2,v_3, v_4)$, $V_2 = (v_5, v_6,v_7, v_8)$, and the voters have the following preferences:For $k=2$ Greedy-CC picks $\{a,b\}$ in both $E_1$ and $E_2$. However, in $E_1 + E_2$ it picks *c* in the first iteration (as *c* is the unique Borda winner in $E_1+E_2$), which means that $\{a, b\}$ cannot be a winning committee in $E_1+E_2$.

For Greedy-Monroe, we let $X=\{x_1, \ldots , x_{20}\}$, $C = \{a, b\}\cup X$, and consider two elections $E_1 = (C,V_1)$ and $E_2 = (C,V_2)$ over the candidate set *C*, where $V_1 = (v_1, v_2, v_3, v_4)$, $V_2 = (v_5, v_6, v_7, v_8)$, and the voters have the following preferences:For $k=2$, Greedy-Monroe chooses $\{a,b\}$ as the unique winning committee in both elections, but for $E_1+E_2$ it chooses *a* and the voters in $V_1$ in the first iteration and then $x_1$ and the voters in $V_2$ in the second iteration, and hence $\{a, x_1\}$ is the unique winning committee. $\square$

We now consider homogeneity. Naturally, committee scoring rules are homogeneous because consistency implies homogeneity. For other rules, the situation is more complex.

### Proposition 13

Both $\ell _{\min }$-CC and Greedy-CC satisfy homogeneity.

We omit the formal proof of Proposition 13; for both rules, the result follows immediately from the definition of the rule.

Interestingly, none of the variants of Monroe’s rule is homogeneous.

### Proposition 14

None of $\ell _1$-Monroe, $\ell _{\min }$-Monroe, and Greedy-Monroe is homogeneous.

### Proof

Consider an election $E=(C, V)$ over the candidate set $C = \{a,b,c,d\}$, where $V=(v_1, v_2, v_3)$ and the voters have the following preferences:Let $k = 2$. For each of $\ell _1$-Monroe, Greedy-Monroe and $\ell _{\min }$-Monroe the unique winning committee of size 2 for *E* is $\{a,c\}$. However, for 2*E* under each of there rules the set of winning committees of size 2 includes $\{a,b\}$. $\square$

On the positive side, if the number of voters is divisible by the target committee size, then $\ell _1$-Monroe and $\ell _{\min }$-Monroe are homogeneous. In essence, this means that these variants of Monroe’s rule fail homogeneity because of rounding imposed by Monroe’s criterion. One solution would be to clone each voter *k* times when seeking a committee of size *k*. We do not consider this modification of Monroe’s rule here, as it is fundamentally incompatible with the idea of fully proportional representation: effectively, under this approach a voter would be fractionally represented by multiple candidates, and it is not clear how to interpret such assignments. However, it would be interesting to see how the satisfaction of a committee elected in this way compares to that of a committee elected without cloning.

### Proposition 15

Both $\ell _1$-Monroe and $\ell _{\min }$-Monroe satisfy homogeneity as long as the number of voters *n* in the election is divisible by the target committee size *k*.

### Proof

Let ${{\mathcal {R}}}\in \{\ell _1$-Monroe, $\ell _{\min }$-Monroe$\}$. Fix an election $E = (C,V)$, and let *k* be a positive integer that divides $n=\Vert V\Vert$. We will show that ${{\mathcal {R}}}(E,k) = {{\mathcal {R}}}(tE,k)$ for every positive integer *t*.

For each $t>0$, let $V_1, \ldots , V_t$ be *t* copies of *V* so that $tV = V_1 + \cdots + V_t$; we assume that within each $V_\ell$, $\ell \in [t]$, voters are listed in the same order. For each $\ell \in [t]$, $i\in [n]$, we write $v_{i,\ell }$ to denote the *i*-th voter in $V_\ell$.

Given a positive integer *t*, consider an assignment $\varPhi : tV \rightarrow C$ that satisfies Monroe’s criterion, i.e., $\Vert \varPhi (tV)\Vert =k$ and each candidate in $\varPhi (tV)$ is assigned to $\frac{nt}{k}$ voters. We say that $\varPhi$ is *t*-*uniform* if it satisfies Monroe’s criterion for each $\ell \in [t]$, i.e., if each candidate in $\varPhi (tV)$ is assigned to $\frac{n}{k}$ voters in $V_\ell$ for each $\ell \in [t]$. We will now show that every assignment $\varPhi : tV\rightarrow C$ satisfying Monroe’s criterion can be transformed into a *t*-uniform assignment with the same societal satisfaction. The proof is by induction on *t*. Our claim trivially holds for $t=1$. Thus, suppose that $t>1$ and our claim has been proved for each $t'\in [t-1]$; we will show that it holds for *t*. Let $W=\varPhi (tV)$.

Set ${{\mathrm {rep}}}(i) = \{ \varPhi (v_{i,\ell }) \mid \ell \in [t] \}$: the set ${{\mathrm {rep}}}(i)$ consists of the candidates assigned to one of the *t* “copies” of the *i*-th voter in $V_1$. Observe that if we swap the values of $\varPhi$ for $v_{i,\ell }$ and $v_{i,\ell '}$ for some $\ell , \ell '\in [t]$ then we get an assignment with the same societal satisfaction. In particular, we can modify $\varPhi$ so as to assign an arbitrary member of ${{\mathrm {rep}}}(i)$ to $v_{i, 1}$. We now show how to use this idea to transform $\varPhi$ into an assignment $\varPhi '$ that assigns each candidate from *W* to exactly $\frac{n}{k}$ voters from $V_1$. We build the following bipartite graph *B* with parts $V_1$ (voter vertices) and $W'$ (candidate vertices):

1. The voter vertices are exactly the voters from $V_1$.
2. For each $w \in W$ the set $W'$ contains $\frac{n}{k}$ candidate vertices $w^1, \ldots , w^{\frac{n}{k}}$, which we will call *clones* of *w*.
3. For each $v_{i, 1} \in V_1$ and each $w\in {{\mathrm {rep}}}(i)$ there are edges between the voter vertex $v_{i, 1}$ and each of the candidate vertices $w^1, \ldots , w^{\frac{n}{k}}$. There are no other edges in *B*.

Note that $\Vert V_1\Vert =\Vert W\Vert =n$. We will now show that *B* admits a perfect matching. For each subset *U* of voter vertices, let *N*(*U*) denote the set of neighbors of *U*, i.e., the candidate vertices that are connected to some member of *U*. By Hall’s theorem, there is a perfect matching in *B* if and only if $\Vert N(U)\Vert \ge \Vert U\Vert$ for each $U\subseteq V_1$.

Let *U* be an arbitrary subset of voter vertices. By construction, if *N*(*U*) contains one clone of *w*, it contains all of them. Thus $\Vert N(U)\Vert = q\frac{n}{k}$ for some positive integer *q*, and, moreover, there is a set of *q* candidates such that *N*(*U*) consists of clones of these candidates. Hence, in *tV* there is a group of $t\Vert U\Vert$ voters that are assigned to exactly *q* candidates. Since each candidate in *W* is assigned to exactly $t\frac{n}{k}$ voters, we obtain $t\Vert U\Vert \le qt\frac{n}{k}$, or, equivalently, $\Vert U\Vert \le q\frac{n}{k} = \Vert N(U)\Vert$. Thus, the condition of Hall’s theorem is satisfied and hence *B* admits a perfect matching.

Given a perfect matching in *B*, we transform $\varPhi$ as follows: for each $i\in [n]$ we identify a candidate *w* such that $v_{i, 1}$ is matched to $w^u$ for some $u\in [\frac{n}{k}]$ and a voter $v_{i, \ell }$ with $\varPhi (v_{i,\ell }) = w$ (such a voter exists since $w\in {{\mathrm {rep}}}(i)$), and swap the representatives of $v_{i,1}$ and $v_{i,\ell }$ (if $\varPhi (v_{i, 1})=w$, we do nothing). In the resulting assignment, which we denote by $\varPhi '$, each candidate in *W* represents exactly $\frac{n}{k}$ voters in $V_1$.

Now, consider the restriction of $\varPhi '$ to $V'=V_2+\cdots +V_t$. Observe that $\varPhi '(V')=W$ and each candidate in *W* is assigned to exactly $(t-1)\frac{n}{k}$ voters in $V'$. Thus, by the induction hypothesis we can transform the restriction of $\varPhi '$ to $V'$ into a $(t-1)$-uniform assignment $\varPhi ''$ that has the same societal satisfaction. It remains to observe that the assignment $\varPhi ^*:tV\rightarrow W$ given by

$$\begin{aligned} \varPhi ^*(v) = {\left\{ \begin{array}{ll} \varPhi '(v) &{} \quad \text {if } \; v\in V_1 \\ \varPhi ''(v) &{} \quad \text {if } \; v\in V' \end{array}\right. } \end{aligned}$$

is *t*-uniform and has the same societal satisfaction as $\varPhi$. This completes the inductive proof.

We are now ready to show that ${{\mathcal {R}}}(E, k)\subseteq {{\mathcal {R}}}(tE, k)$ for each $t>0$. Consider a committee $W\in {{\mathcal {R}}}(E, k)$, where $E=(C, V)$, and let $\varPhi :V\rightarrow W$ be an assignment of candidates in *W* to voters in *V* that maximizes the societal satisfaction among all assignments that satisfy Monroe’s criterion. Pick $t>0$ and consider an assignment $\varPhi ':tV\rightarrow W$ given by $\varPhi '(v_{i, \ell })=\varPhi (v_i)$ for each $i\in [n]$, $\ell \in [t]$. If $W\not \in {{\mathcal {R}}}(tE, k)$ then there is another committee $W'$ of size *k* and an assignment function $\varPhi ':tV\rightarrow W'$ that satisfies Monroe’s criterion and provides a higher societal satisfaction than $\varPhi$ does. By the argument above, we can assume that $\varPhi '$ is *t*-uniform. But then there exists an $\ell \in [t]$ such that the restriction of $\varPhi '$ to $V_\ell$ provides a higher societal satisfaction to voters in $V_\ell$ than $\varPhi$ does, a contradiction with $W\in {{\mathcal {R}}}(E, k)$. Thus, $W\in {{\mathcal {R}}}(tE, k)$.

Finally, we will show that ${{\mathcal {R}}}(tE, k)\subseteq {{\mathcal {R}}}(E, k)$ for each $t>0$. Fix $t>0$ and suppose that $W\in {{\mathcal {R}}}(tE, k)$. Let $\varPhi :tV\rightarrow W$ be an assignment of candidates in *W* to voters in *tV* that maximizes the societal satisfaction among all assignments that satisfy Monroe’s criterion. Again, we can assume that $\varPhi$ is *t*-uniform. Together with the optimality of $\varPhi$, this means that each set of voters $V_\ell$, $\ell \in [t]$, experiences the same societal satisfaction under $\varPhi$. Indeed, if that was not the case, i.e., $V_\ell$ had a higher satisfaction under $\varPhi$ than $V_j$ did for some $\ell , j\in [t]$, the assignment $\varPhi ':tV\rightarrow W$ with $\varPhi '(v_{i, j})=\varPhi (v_{i, \ell })$ for $i\in [n]$, $\varPhi '(v)=\varPhi (v)$ for $v\not \in V_j$ would satisfy Monroe’s criterion and provide a higher societal satisfaction than $\varPhi$ did, a contradiction. Now, suppose for the sake of contradiction that $W\not \in {{\mathcal {R}}}(E, k)$. Then there is another committee $W'$ of size *k* and an assignment $\varPhi ':V_1\rightarrow W'$ that satisfies Monroe’s criterion and provides a higher societal satisfaction to voters in $V_1$ than $\varPhi$ does. But then consider the assignment $\varPhi _t:tV\rightarrow W'$ given by $\varPhi _t(v_{i, \ell })=\varPhi '(v_{i, 1})$ for each $\ell \in [t]$. By construction, it satisfies Monroe’s criterion and provides a higher societal satisfaction to voters in *tV* than $\varPhi$ does, a contradiction with $W\in {{\mathcal {R}}}(tE, k)$. Thus, $W\in {{\mathcal {R}}}(E, k)$. $\square$

### Proposition 16

Greedy-Monroe fails homogeneity even if the target committee size divides the number of voters.

### Proof

Consider an election $E = (C,V)$ over the candidate set $C = \{a,b,c,d\}$, where $V = (v_1, \ldots , v_6)$, and the voters have the following preferences:For $k=2$ Greedy-Monroe outputs $\{a,b\}$ and $\{a,c\}$. Indeed, in the first iteration it picks *a* or *b*. If it picks *a*, it can assign it to $(v_1,v_2,v_3)$, $(v_1,v_3,v_5)$, or $(v_1,v_3,v_6)$. Depending on this choice, in the second iteration it picks either *c* or *b*. If it picks *b* in the first iteration, it has to choose *a* in the second iteration.

Now consider the election 2*E*. For each $i\in \{1,\ldots , 6\}$, $j\in \{1, 2\}$ let $v_{i, j}$ denote the *i*-th voter in the *j*-th copy of *E*. In the first iteration, Greedy-Monroe may pick *a* and assign it to $(v_{1, 1},v_{1, 2},v_{2, 1},v_{3, 1},v_{3, 2},v_{5, 1})$. The remaining votes are:

$$\begin{aligned} v_{6, 1}:&d \succ a \succ c \succ b, \quad v_{2, 2}:b \succ a \succ d \succ c, \quad v_{5, 2}:c \succ a \succ d \succ b, \\ v_{4, 1}:&b \succ c \succ d \succ a, \quad v_{4, 2}:b \succ c \succ d \succ a, \quad v_{6, 2}:d \succ a \succ c \succ b. \end{aligned}$$

The unique Borda winner of this election is *d*, so Greedy-Monroe picks *d* in the second iteration. This means that $\{a,d\}$ is a winning committee in 2*E*, and hence Greedy-Monroe is not homogeneous. $\square$

Proposition 16 relies heavily on parallel-universes tie-breaking. It is possible to refine the intermediate tie-breaking procedure of Greedy-Monroe so that it becomes homogeneous when *k* divides $\Vert V\Vert$. We omit the details here.

## Related work

Having characterized a number of natural committee selection rules with respect to the axioms we proposed, we are now in a position to discuss how our results and approaches compare to prior work. The literature on the properties of committee selection rules is still somewhat sparse (at least compared to the body of work on single-winner rules), and it is scattered among different fields of research, ranging from behavioral science, through political science and social choice theory, to computer science. Here we review papers that are most similar in spirit to our work.

In a closely related paper, Felsenthal and Maoz (1992) consider four *k*-choice functions: the Plurality rule (i.e., in our terminology, the SNTV rule), the Approval rule,^4^ the Borda rule (i.e., *k*-Borda), and STV. They adapt a range of single-winner normative properties to the committee selection setting and study them in the context of these rules. In contrast with our work, they consider axioms motivated by the Condorcet principle, while we focus on axioms that capture ideas related to proportional representation. However, both papers consider monotonicity, though our analysis is somewhat more detailed in that we study two variants of this property (candidate monotonicity and non-crossing monotonicity), as well as committee monotonicity (Felsenthal and Maoz use the term ‘continuity’) and consistency (also known as reinforcement). Interestingly, consistency also plays an important role in the work of Bock et al. (1998) on consensus-based multiwinner rules. Debord (1993) also introduced several axioms for multiwinner rules. These axioms, however, are geared towards the rules that elect what he calls *k*-elites, which are multiwinner analogs of Condorcet winners.

Young’s consistency-based characterization of scoring rules, as well as his characterization of the Borda rule, have already inspired researchers working on committee selection rules. For example, Debord (1992) extended Young’s characterization of Borda to the case of *k*-Borda, and very recently Skowron et al. (2016b) extended Young’s ideas to characterize the class of committee scoring rules. Faliszewski et al. (2016b) study a subclass of committee scoring rules which they call top-*k*-counting rules, and provide an axiomatic characterization of the Bloc rule, which belongs to this class. Faliszewski et al. (2016a) propose a hierarchy of committee scoring rules.

Skowron (2015) offers a different perspective on committee selection rules. He assumes that the elected committee will have to make several decisions, i.e., vote on a number of issues, and that voters evaluate committees based on the final outcome of this two-stage process (which the voter can predict to a certain extent). The performance of a committee selection rule then depends on the single-winner voting rule that the committee will use to make its decisions. In this setting, several voting rules that we consider, such as $\ell _1$-CC, emerge as optimal voting rules for appropriate assumptions about voters’ preferences.

There is also a considerable amount of work on committee selection rules in the framework of approval voting. Kilgour (2010) describes a number of approval-based voting rules that elect a committee of fixed size and establishes some of their basic properties. Kilgour and Marshall (2012) give an excellent survey of approval-based committee selection rules and propose some new ones. A recent paper by Aziz et al. (2017) is conceptually similar to our work: there, the authors consider a number of popular approval-based committee selection rules, introduce a new axiom for such rules—similar in spirit to Dummett’s solid coalitions property and called *justified representation*—and check which of the rules in their list satisfy it.

We conclude our discussion of related work by mentioning a very recent paper by Elkind et al. (2017), where the authors visually present aggregated outcomes of a number of multiwinner voting rules. Their experimental results confirm our theoretical predictions, and also reveal some effects that are hard to discover by purely theoretical means.

**Table 1:** Summary of results

Rule	Committee	Solid	Consensus	Unanimity	Monotonicity	Homogeneity	Consistency
	Monotonicity	Coalitions	Committee				
STV	$\times$	$\surd$ ($\diamond$)	$\surd$ ($\diamond$)	Strong	$\times$	$\surd$($\heartsuit$)	$\times$
SNTV	$\surd$	$\surd$	$\surd$	Weak	C+NC	$\surd$	$\surd$
Bloc	$\times$	$\times$	$\times$	Fix maj.	C+NC	$\surd$	$\surd$
*k*-Borda	$\surd$	$\times$	$\times$	Strong	C+NC	$\surd$	$\surd$
$\ell _1$-CC	$\times$	$\times$	$\surd$	Weak	C	$\surd$	$\surd$
$\ell _{\min }$-CC	$\times$	$\times$	$\surd$	Weak	C	$\surd$	$\times$
Greedy-CC	$\surd$	$\times$	$\times$	Weak	$\times$	$\surd$	$\times$
$\ell _1$-Monroe	$\times$	$\times$	$\surd$	Strong	$\times$	$\surd$ ($\clubsuit$)	$\times$
$\ell _{\min }$-Monroe	$\times$	$\times$	$\surd$	Strong	$\times$	$\surd$ ($\clubsuit$)	$\times$
Greedy-Monroe	$\times$	$\surd$	$\surd$	Strong	$\times$	$\surd$ ($\spadesuit$)	$\times$

$\varvec{\surd }$ and $\varvec{\times }$ indicate that the rule has/does not have the respective property. C means candidate monotonicity and NC means non-crossing monotonicity (C+NC means having both properties). “Fix maj.” in the Unanimity column for Bloc means that Bloc is not just unanimous in the strong sense, but also satisfies the fixed majority property, which is stronger (none of the other rules satisfy it). The properties marked with ($\diamond$) hold for STV when $n \ge k(k+1)$; the property marked with (${\heartsuit }$) requires STV to use non-rounded Droop quota and fractional votes. The properties marked with (${\clubsuit }$) hold if *n* is divisible by *k* and $({\spadesuit }$) in addition requires a specific intermediate tie-breaking rule

## Conclusions

We formalized a number of natural properties of committee selection rules and conducted a comprehensive comparison of ten prominent committee selection rules with respect to these properties. Our results are summarized in Table 1. In the course of our study, we identified two natural families of committee selection rules—best-*k* rules and committee scoring rules—and related these families of rules to the properties we consider. In particular, we characterized best-*k* rules as the only rules that satisfy committee monotonicity, identified simple conditions on committee scoring rules that ensure the consensus committee property or non-crossing monotonicity, and showed that all committee scoring rules satisfy weak unanimity, candidate monotonicity, and consistency.

We believe that the results in this paper improve our understanding of applicability of various multiwinner rules to particular tasks. For example, we see that best-*k* rules are well-suited for selecting a group of finalists in a competition, whereas STV and rules based on Monroe’s criterion ($\ell _1$-Monroe, $\ell _{\min }$-Monroe, and Greedy-Monroe) seem to be more appropriate for applications that require proportional representation (e.g., parliamentary elections).

In this context, Greedy-Monroe is particularly interesting. It was introduced as an approximation algorithm for $\ell _1$-Monroe, but, according to our criteria, it is more attractive than the original rule. Therefore, we believe that Greedy-Monroe should be treated as a full-fledged voting rule. In contrast, Greedy-CC, which was designed as an approximation algorithm for $\ell _1$-CC, does not appear to perform well in our comparison. Indeed, it fails to satisfy the solid coalitions property (like $\ell _1$-CC, but unlike Greedy-Monroe) or the consensus committee property (unlike every other rule that focuses on some form of proportional representation). The latter fact can be seen as a consequence of Greedy-CC satisfying committee monotonicity (which we argued to be incompatible with the goal of proportional representation). Given this comparison, it is tempting to simply use Greedy-Monroe in place of Greedy-CC. However, perhaps a better idea would be to modify Greedy-Monroe by allowing it to decide how many voters to consider in each iteration. We believe that studying such variant of Greedy-Monroe is an important research direction.

Finally, our results regarding SNTV are quite interesting. This is the only separable committee scoring rule that is also representation-focused. Moreover, it satisfies almost all axioms considered in our work, except strong unanimity. One explanation for this fact is that it can be viewed as a ‘budget-constrained’ variant of the Tullock rule (Tullock 1967); this is the rule that simply picks all the candidates that are ranked first by at least one voter. The Tullock rule possesses nice axiomatic properties, but cannot be used to output a committee of a given size. Of course, since SNTV ignores each voter’s preferences beyond the top candidate, it may return highly counterintuitive results in many cases. For instance, it is extremely indecisive when voters are unanimous: in this case, it may return any committee containing the candidate ranked first by all voters (in particular, it may select a committee that does not include the candidate ranked second by all voters). Also, if the opinions are widely divided, a small group of voters can easily coordinate their efforts to get their preferred candidate elected, even if this candidate is ranked last by all other voters. These flaws of SNTV severely restrict the range of scenarios where it can be used.

## Footnotes

1 For example, the upper house of the Parliament of Australia uses a variant of STV; a variant of SNTV is used, e.g., in Puerto Rico.

2 For several committee scoring rules (including SNTV, Bloc, and *k*-Borda), Diss and Doghmi (2016) consider the probability that a Condorcet committee in the sense of Gehrlein (1985) is elected, provided that one exists.

3 However, there is a variant of Dummett’s condition known as *Droop Proportionality Criterion*, which uses the Droop quota in place of $\frac{n}{k}$ (Woodall 1994); STV can be shown to satisfy this criterion, and hence it satisfies Dummett’s original condition when the Droop quota is smaller than $\frac{n}{k}$.

4 This rule is not preference-based.
